# Operational Risk Assessment Tool for Evaluating *Leishmania infantum* Introduction and Establishment in the United States through Dog Importation^1^

**DOI:** 10.3201/eid3012.231084

**Published:** 2024-12

**Authors:** David R. Marquez, Anne Straily, Keeve Nachman, Douglas E. Norris, Meghan F. Davis, Christine A. Petersen

**Affiliations:** US Army Veterinary Services, Aberdeen Proving Ground, Maryland, USA (D.R. Marquez); Johns Hopkins University, Baltimore, Maryland, USA (D.R. Marquez, K. Nachman, D.E. Norris, M.F. Davis); Centers for Disease Control and Prevention, Atlanta, Georgia, USA (A. Straily); University of Iowa, Iowa City, Iowa, USA (C.A. Petersen)

**Keywords:** Leishmania infantum, leishmaniosis, parasites, vector-borne diseases, zoonoses, dog importation, import risk assessment, One Health, United States

## Abstract

International pet travel and commercial operations have increased animal disease importation risks, including for *Leishmania infantum*, a deadly parasite of humans and domestic dogs. Collaborating as an interdisciplinary working group, we developed an operational tool for veterinary and public health practitioners to assess and manage *L. infantum* risk in dogs imported to the United States. Overall risk varies by dog, human, and geographic factors but could be high without proper controls. We determined dog risk management strategies should include application of sand fly insecticides and repellents, sterilization, and treatment. US public health authorities can use a One Health approach to manage *L. infantum* importation risks via infected dogs.

International pet travel and importation of breeding and rescue animals have increased substantially, spreading diseases beyond established geographic distributions (*1*–*3*). The Centers for Disease Control and Prevention (CDC) estimates 1 million dogs cross US borders annually (*4*). Despite cooperation among federal agencies, <0.3% of dogs receive animal import certifications, and most do not receive adequate infectious disease screening before entering the United States (*2*,*4*,*5*).

Unregulated dog importation into the United States from leishmania-endemic countries could continue to introduce *Leishmania infantum*, one of the world’s deadliest tropical parasites (*6*). *L. infantum* is a protozoal parasite that causes zoonotic visceral leishmaniasis (ZVL) in humans and canine leishmaniosis (CanL) in dogs. Dogs are the primary reservoir hosts in endemic areas, which include southern Europe, North Africa, the Middle East, Central Asia, China, and South and Central America (*7*). Annually, an estimated 10,000–18,000 ZVL cases are reported globally, and disease is usually fatal without treatment (*8*). Domestically acquired human ZVL cases have not been identified within the United States, but CanL has been found in US hunting dogs across 60 kennels and 28 states since the early 2000s (*9*,*10*). However, the parasite is spreading because of globalization and climate change (*11**–**13*). Over the past 20 years, dog movements resulted in >1,400 nonautochthonous canine *Leishmania* spp. infections in multiple countries (*11*). Almost all *L. infantum–*positive nonhunting dogs in the United States have history of travel to *L. infantum*–endemic areas (*14*).

*L. infantum* parasites are prevalent among hunting dogs in several US states, but few states have formal disease surveillance and control programs (*15*). The number of *L. infantum*–infected dogs imported into the United States is unknown, as is the risk importation poses for establishing autochthonous ZVL and further spread of CanL among US nonhunting dogs. We devised an operational risk assessment tool (ORAT) to address the probability of importing *L. infantum*–infected dogs into the United States from endemic areas, the probability of vectored transmission via sand flies among US dogs, and potential impacts of autochthonous *L. infantum* parasite transmission on canine and human health. The tool provides public and animal health officials with a risk assessment framework and evidence-based mitigation recommendations when importing potentially infected dogs from *L. infantum*­–endemic countries.

## Risk Assessment Methodology

An interdisciplinary working group whose members have backgrounds in veterinary epidemiology, infectious diseases, public health, vector ecology, and risk sciences, developed this ORAT by using established qualitative risk assessment frameworks (*16*–*21*). Risk assessment occurs in 3 phases. First, the hazard is identified along with key assumptions needed for characterizing the hazard and risks that the hazard will occur. Next, necessary steps for the hazard to occur are defined as the entry and exposure assessments, and the probability of each step occurring are characterized into qualitative risk categories, such as very low, low, moderate, and high. Finally, the consequence is assessed, and a final risk is determined on the basis of probability and impacts. 

**Figure 1:**
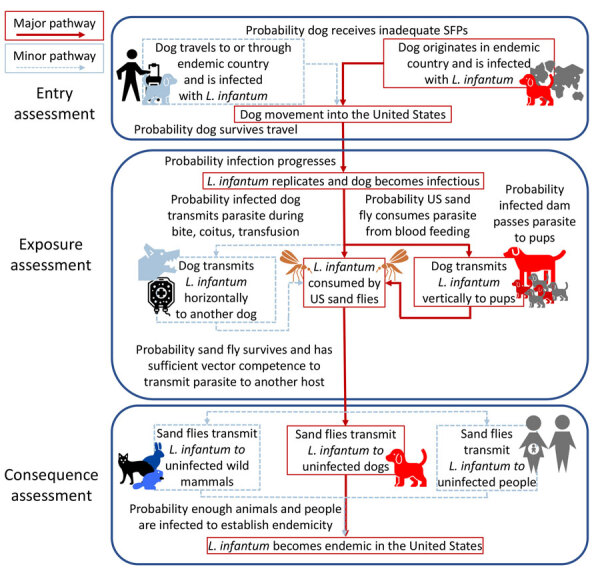
Scenario tree used to develop an operational risk assessment tool for evaluating *Leishmania infantum* introduction and establishment in the United States through dog importation. SFPs, sand fly preventatives.

**Figure 2:**
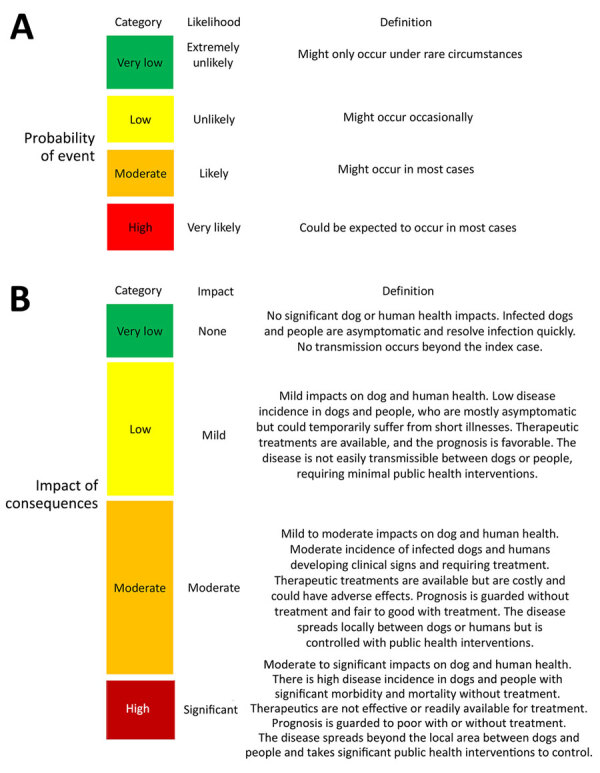
Probability and impact categories for an operational risk assessment tool for evaluating *Leishmania infantum* introduction and establishment in the United States through dog importation. Modified from Food and Agriculture Organization of the United Nations (*20*).

**Figure 3:**
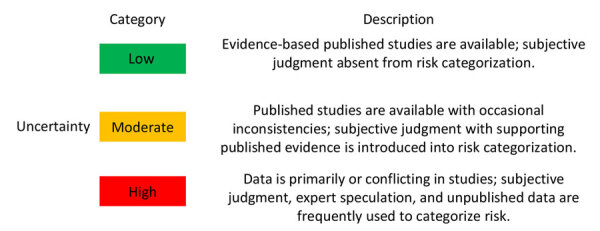
Uncertainty categories for an operational risk assessment tool for evaluating *Leishmania infantum* introduction and establishment in the United States through dog importation. Modified from Food and Agriculture Organization of the United Nations (*20*).

In this ORAT, the entry assessment considers the probability of importing *L. infantum*–infected dogs into the United States, the exposure assessment estimates the potential for vector transmission by domestic sand fly species, and the consequence assessment considers the impact of autochthonous *L. infantum* transmission on dog and human health (Figure 1). This risk assessment used expert opinion combined with a review of current available evidence at each phase. We describe the rationale for each probability and the uncertainty of the evidence (Figures 2, 3).

## Results

### Hazard Identification

**Figure 4:**
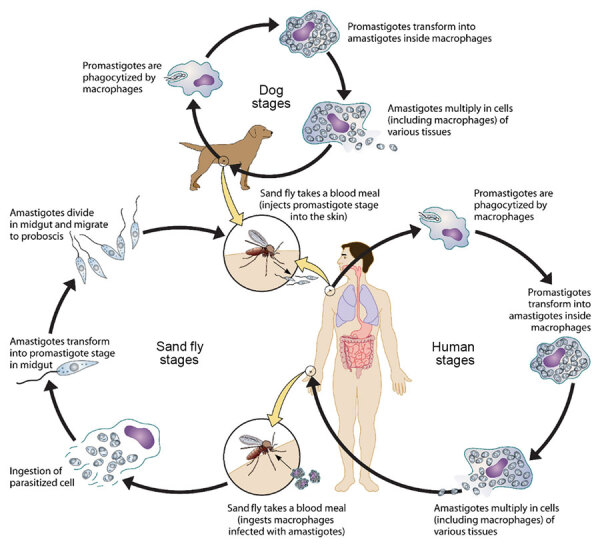
Parasite lifecycle used to develop an operational risk assessment tool for evaluating *Leishmania infantum* introduction and establishment in the United States through dog importation. Reproduced from Esche et al. (*29*); used by permission of the American Society for Microbiology.

The biologic hazard is *L. infantum*, which is primarily transmitted by female sand flies (genus *Phlebotomus* or *Lutzomyia*), but vertical and horizontal transmission have been increasingly documented in dogs (*10*,*22*–*27*). *L. infantum* parasites evade immune recognition and multiply within macrophages in major organs, such as the skin, liver, spleen, and bone marrow (*28*,*29*) (Figure 4). Inflammatory cytokine and immunoglobulin production results in nonspecific clinical signs such as fever, weight loss, and organomegaly. Dogs develop prominent dermatological lesions and glomerulonephritis, which greatly reduces prognosis in advanced stages (*28*,*30*). Immunocompromised persons are at greatest risk for disease progression, particularly persons with HIV (*8*), pregnant women (*31*), children (*8*), and intravenous drug users (*32*). Poor nutrition, co-infections, and stress may exacerbate CanL and ZVL disease progression (*29*,*33*).

*L. infantum*–infected dogs are highly prevalent in endemic areas, are difficult to identify and treat, and pose disease transmission risks to other animals and humans. *L. infantum* parasites are prevalent among hunting dogs in several US states but otherwise are not endemic. In the United States, *L. infantum* parasites are passed from infected dams to pups without apparent vector transmission, making vertical transmission the dominant mode of disease spread (*10*,*23*,*34*). Despite experimental studies confirming parasite transmission from sand flies fed on infected hunting dogs (*35*), no evidence of vectored *L. infantum* transmission among US dogs or humans has been reported (*9*,*10*,*35*). The United States has >14 sand fly species, and 3 are known vectors of *Leishmania* parasites: *Lutzomyia anthophora*, *Lu. diabolica*, and *Lu. shannoni* (*36*–*38*). *Lu. anthophora* and *Lu. diabolica* sand flies are known vectors of *L. mexicana* parasites (*37*,*39*–*41*) but not of other *Leishmania* spp. in the United States. Studies of *Lu. shannoni* sand flies raise concern that it could be a permissive vector for *L. infantum* (*35*,*42*).

US importation of asymptomatic, infected dogs remains a likely route of *Leishmania* parasite entry (*14*). For this risk assessment tool, we considered *L. infantum* a substantial hazard in domestic dogs imported from endemic areas into the United States.

### Key Assumptions Used in ORAT Development

Key assumptions we used for developing the ORAT were reservoirs, vectors, and preventive measures. For reservoirs, we considered dogs imported into the United States to represent the general US dog population that are equally susceptible to *L. infantum* infection and are equally infectious to sand flies (i.e., not super spreaders) (*43*,*44*). We considered vectors to include *Lu. shannoni* sand flies, which could act as permissive vectors for *L. infantum* transmission among dogs. For preventive measures, we considered sand fly preventatives to include insecticidal and repellent collars, sprays, and topicals experimentally shown to reduce sand fly feeding and lifespans. For this ORAT, we assumed that US sand flies are susceptible to commercially available SFPs and products are correctly applied on dogs according to manufacturer’s guidelines.

### Entry Assessment

**Figure 5:**
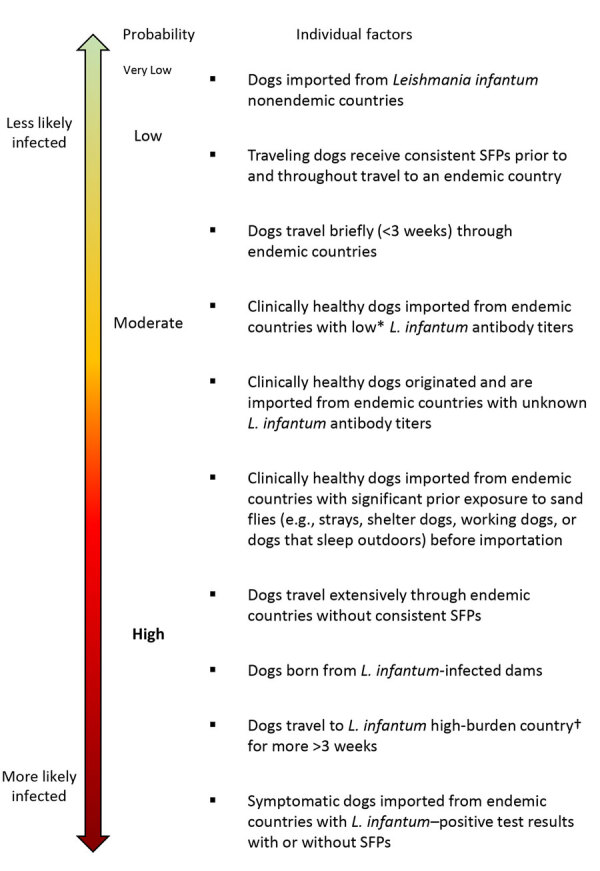
Probability scale used to develop an operational risk assessment tool for evaluating *Leishmania infantum* introduction and establishment in the United States through dog importation. *Negative serologic titers are typically those <1:40, but titer thresholds vary by laboratory. Titer values 1–2-fold higher than reference thresholds should be considered low titers and values >2-fold the reference should be considered a high titer (*45*). †Endemic and high burden countries are listed elsewhere (Appendix). SFPs, sand fly preventatives.

The entry assessment describes biologic pathways needed for importation of an infected dog to introduce *L. infantum* parasites into the United States (Figure 5) and estimates the probability of that process occurring (*19*). Entry assessment rationale considers that prevalence estimates of infected dogs in endemic countries range from 3% to 24% but can exceed 70% in highly endemic areas (*7*,*46*–*48*). Officials tasked with assessing risk (risk assessors) posed by an imported dog should consider the dog’s country of origin and that country’s endemicity status (*19*) (Appendix), in addition to individual factors, such as time spent in endemic areas, SFP use, and the dog’s occupation, to determine the dog’s infection probability during the entry assessment.

Dogs traveling to *L. infantum*–endemic areas for <3 weeks are at low risk (<1%) for infection, particularly if prophylactically treated with SFPs (*6*,*49*–*50*; Appendix reference *51*). Infection risk increases with time spent in endemic areas, particularly for dogs outdoors during sand fly season (*7*). *L. infantum* vaccines available in Europe and Brazil have varying levels of protection but may reduce infection rates in healthy seronegative dogs (Appendix reference *52*).

Most *L. infantum*–infected dogs are asymptomatic, creating challenges assessing importation risks in healthy, traveling pets. Serologic diagnostic tests such as indirect fluorescent antibody tests (IFAT) and ELISA are widely used but may cross-react with antibodies to *Trypanosoma cruzi* parasites (Appendix reference *53*). Quantitative *L. infantum* serologic titers >1:320 are often associated with high parasitism and CanL in dogs with clinically suspected infections (Appendix reference *54*). Low *L. infantum* titers indicate parasite exposure but not necessarily active infection (*33*; Appendix reference *55*). Risk assessors should carefully weigh the dog’s clinical signs, clinicopathologic abnormalities, and serologic status during entry assessment.

Properly applied SFPs decrease sand fly feeding and survival, drastically reducing risk for *L. infantum* transmission in both endemic and nonendemic areas (Appendix references *51,56–61*). Many pyrethroid topical products have 90% insecticidal and repellent efficacy of <4 weeks duration post application (Appendix reference *56*). Dogs treated with deltamethrin or flumethrin collars or fluralaner oral insecticides were protected from sand fly bites for >5 months (Appendix reference *61–65*). Newer generation insecticides, such as spinosad and isoxazolines, may be effective for preventing sand fly bites but lack clinical evidence supporting their use to prevent CanL (Appendix references *56,66,67*). Natural compounds (e.g., neem oil and citronella) have limited insecticidal and repellency efficacy and are not recommended as SFPs for dogs (Appendix references *68–70*).

A dog’s lifestyle or occupation greatly influences its risk of acquiring infectious diseases (Appendix reference *71*). Stray and shelter dogs in endemic countries are exposed to many zoonotic parasites, including *L. infantum*, and pose an elevated infection risk compared with companion dogs (*48*; Appendix references *72,73*). Working and companion dogs with frequent outdoor exposure are at increased risk for sand fly bites (*13*; Appendix references *71,74–76*). Many governmental agencies provide SFPs for working dogs to reduce sand fly exposure, greatly reducing *L. infantum* infection rates in that cohort (Appendix references *71,74,77*).

### Entry Assessment Uncertainty Level—Low

Sufficient studies are available to characterize the entry assessment, with few discrepancies. On average, dogs develop *L. infantum* antibodies 5 months after infection (Appendix reference *54*). Generally, IFAT, the diagnostic test used most often in the veterinary field (Appendix reference *78*), has a high sensitivity (≈90%) in symptomatic and lower sensitivity (<40%) in asymptomatic dogs in endemic areas (Appendix reference *79*). That sensitivity and the prolonged incubation period limit the utility of testing dogs for *L. infantum* infection before importation. A negative serologic result does not rule out subclinical infections in dogs (*33*,*45*); monitoring for CanL after import should continue regardless of serologic status before entry.

SFP use in *L. infantum–*endemic areas effectively reduces CanL incidence, particularly in working, stray, and shelter dogs. However, studies of different commercial formulations show variations in duration of protection against sand fly bites (Appendix references *65,80*). Furthermore, SFPs are never 100% effective and often show substantial decreases in insecticidal activity before product reapplication. For instance, after application, many monthly ectoparasiticidal topicals lose 75% of sand fly insecticidal activity by 21 days (Appendix references *81,82*). Appropriate SFP use is also difficult to monitor because it is the responsibility of the dog’s owner or caretaker.

Vaccines used in Brazil and Europe are likely to prevent clinical disease progression, but reports conflict as to whether vaccines prevent CanL infections in endemic areas (*6*; Appendix references *52,83,84*). Further studies are needed before vaccinated dogs can be considered low risk for introducing *L. infantum* (Appendix reference *83*).

### Exposure Assessment

**Figure 6:**
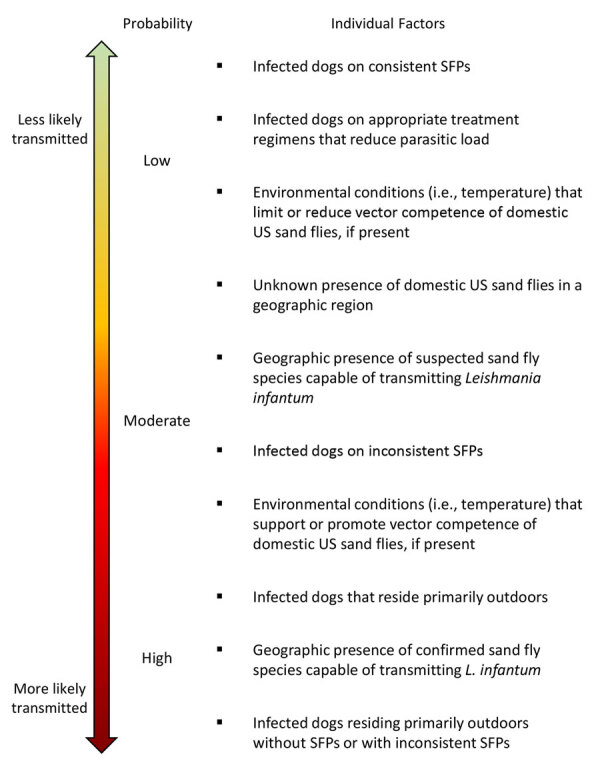
Probability of vectored transmission scale used to develop an operational risk assessment tool for evaluating *Leishmania infantum* introduction and establishment in the United States through dog importation. SFPs, sand fly preventatives.

The exposure assessment describes biologic pathways required for release of *L. infantum* parasites from an infected imported dog and subsequent exposure of humans and other animals in the United States and assesses the likelihood of occurrence (*19*) (Figure 6). The exposure assessment rationale considers that not all sand fly species are competent vectors (i.e., susceptible to infection with *L. infantum* parasites and capable of transmitting to vertebrate hosts) of *Leishmania* spp. (Appendix reference *85*), but many are considered permissive vectors, whereby they support development of Old and New World *Leishmania* parasites but do not further transmit parasites (Appendix references *86,87*). Vector competence and permissiveness are laboratory-defined terms. Few studies have assessed *L. infantum* infection rates of US sand flies, but experimental studies have shown *Lu. shannoni* sand flies support parasite development after feeding on infected dogs and hamsters, making it a permissive vector (*35*,*42*).

*Lu. shannoni* sand flies are widely dispersed across the United States and reported from >17 states (*36*; Appendix references *88–93*). Within its range, *Lu. shannoni* sand fly occurrence is subject to local environmental factors, including precipitation, temperature, habitat availability, and suitable hosts (Appendix reference *94*). The flies feed primarily on mammals, including rodents, livestock, dogs, and deer, and will readily feed on humans (*36*; Appendix references *92,95*).

**Figure 7:**
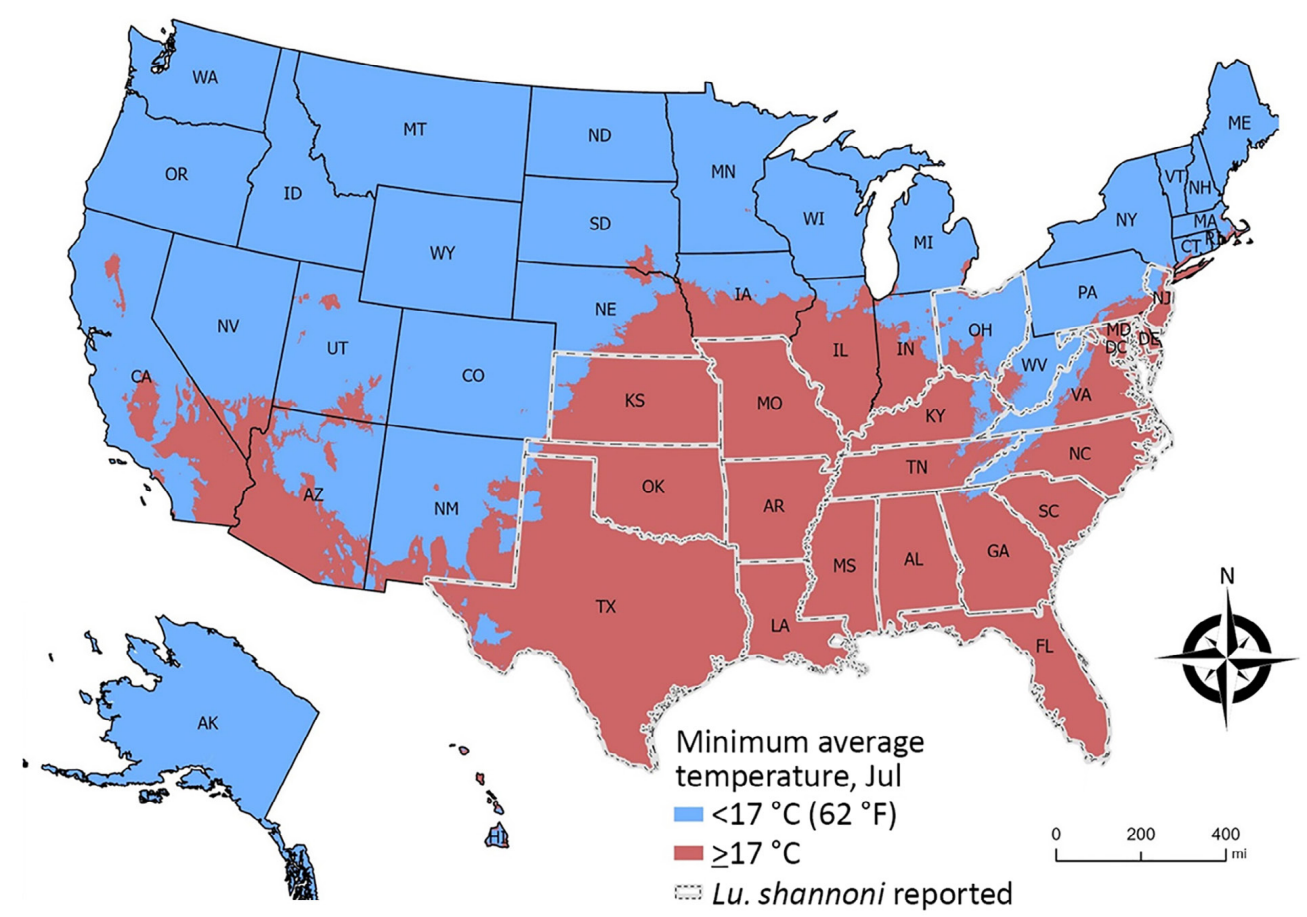
Minimum average July temperatures used to develop an operational risk assessment tool for evaluating *Leishmania infantum* introduction and establishment in the United States through dog importation. Map shows areas in the United States where *Lutzomyia shannoni* sand flies have been reported (white dotted lines); *Lu. shannoni* sand flies could serve as *L. infantum* vectors. Map created with ArcGIS Pro 3.0.2 (Esri, https://www.esri.com) by using NAD 1983 Contiguous USA Albers Projection. Climate data from Worldclim Version 2 (https://worldclim.org) includes average July minimum temperatures during 1970–2000 at 1 km^2^ spatial resolution (Appendix reference 99).

Sand flies develop, feed, and thrive at 20°C–28°C (68°F–82°F) (Appendix references *96–98*), and 17°C (62°F) is considered the minimum sand fly survival temperature (Appendix reference *56*). We devised a risk map by using estimates of potential US sand fly distribution overlayed with average minimum temperatures during peak sand fly season (Figure 7), similar to the *L. infantum* risk map from Europe (Appendix reference *56*). The map does not include humidity, wind, or rain estimates but assesses geographic areas with temperatures above and below 17°C in July, historically the warmest month of the year, to conservatively predict sand fly activity. The map demonstrates states at higher risk for *L. infantum* vector-borne transmission due to reported *Lu. shannoni* sand fly activity (Figure 7, outlined in white dotted lines).

Treatment of CanL in dogs rarely produces a parasitic cure but reduces clinical signs, parasite burden, and risk for transmission to sand flies for >4 months (Appendix references *70,80,100–104*). However, many dogs will relapse and become infectious to sand flies within a year posttreatment (Appendix references *101,105,106*). Second- and third-line therapeutics, such as metronidazole, marbofloxacin, and azole antifungals, improve clinical signs associated with CanL but have not been evaluated for alteration of parasitemia in dogs and did not reduce hepatic parasite loads in mice (Appendix references 107–109).

### Exposure Assessment Uncertainty Level—High 

Climate change has undoubtedly altered sand fly species’ geographic distribution and seasonality, likely increasing the risk for new *L. infantum* infections (Appendix references *86,88,110*). However, the potential magnitude of sand fly habitat expansion within the United States is unknown. Whether US sand fly species, particularly *Lu. shannoni*, can transmit *L. infantum* parasites to uninfected animals or humans in natural settings is also unknown (*42*; Appendix reference *111*). Additional studies are needed to confirm *Lu. shannoni* vector competence in natural settings.

Despite clinical improvement and reduced infectiousness to sand flies after therapy, treated dogs may still harbor viable parasites that sand flies could ingest (Appendix references *112–115*). Not all chemotherapeutic drugs substantially reduce parasitic loads. Chemotherapy may be less effective at reducing parasitic loads in drug-resistant strains, which are reported for meglumine antimoniate and allopurinol in some areas (Appendix references *116,117*). Additional studies in dogs are needed to establish whether azole antifungal drugs further reduce dog infectivity to sand flies (Appendix reference *118*).

**Figure 8:**
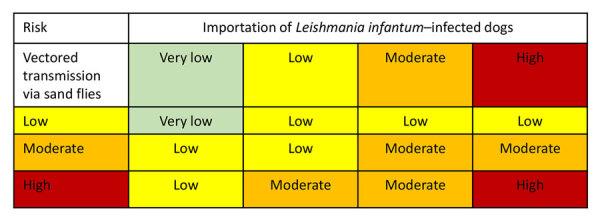
Combined probability matrix for entry and exposure assessments developed from an operational risk assessment tool for evaluating *Leishmania infantum* introduction and establishment in the United States through dog importation. Modified from Wieland et al. (*17*).

Results from numerous studies have identified positive associations between the severity of an infected dog’s clinical and laboratory findings and its infectiousness to sand flies (Appendix references *119–123*). However, clinical scores used to define severity in those studies were highly variable and might not have accounted for all clinicopathologic abnormalities or skin parasite burden, making comparison of patient infectiousness across studies challenging (Appendix reference *124*). Results from more recent studies have established skin parasitic burden as most predictive of transmission and demonstrated that mildly affected dogs were more infectious to sand flies than were severely affected dogs (*44*). When working through the ORAT, the entry and exposure probabilities can be combined in a combination probability matrix that determines conditional probability estimates of *L. infantum* parasite importation via infected dogs, followed by vectorborne transmission in the United States (Figure 8).

### Consequence Assessment

**Figure 9:**
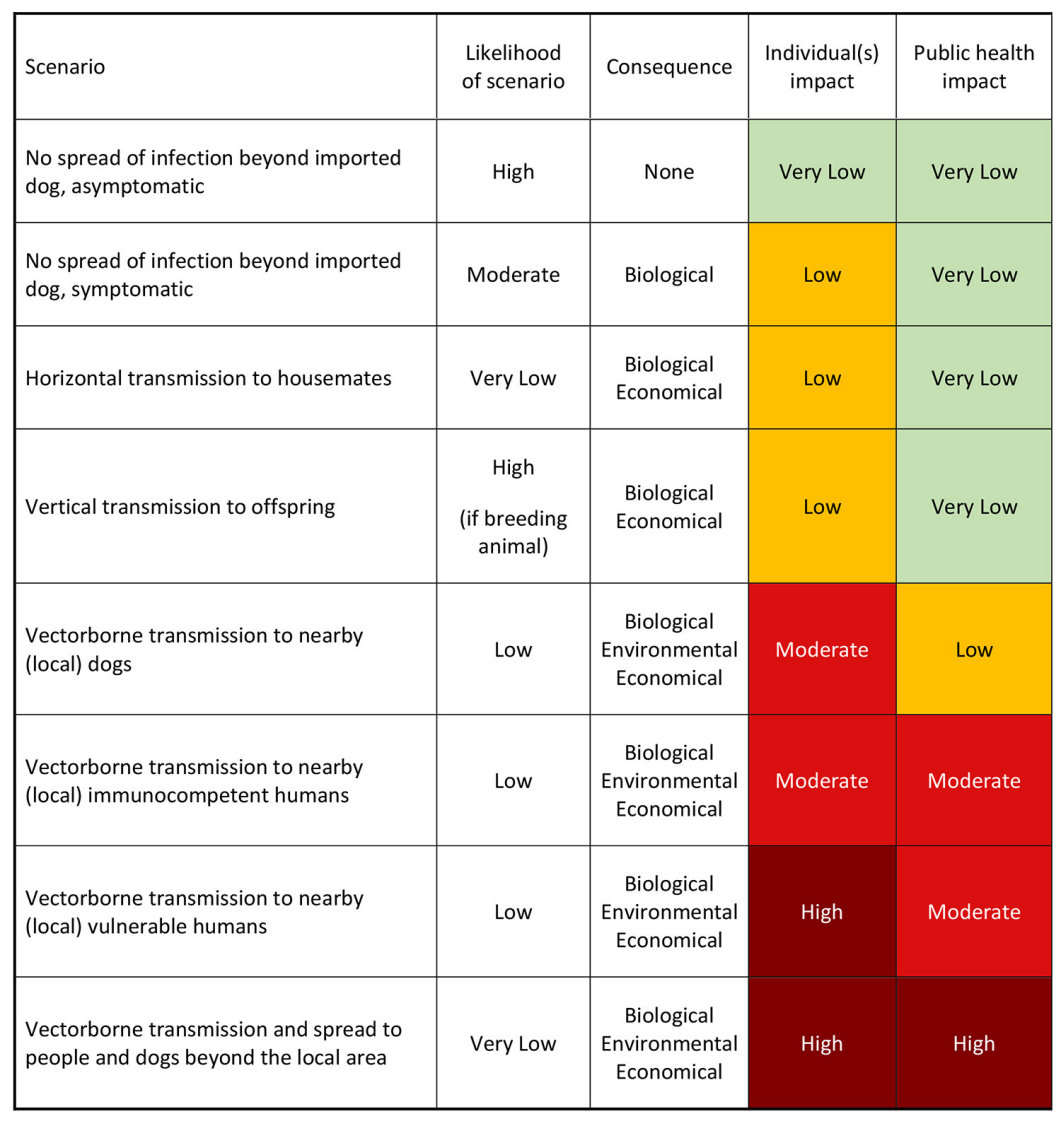
Scenarios and possible impact of autochthonous transmission on dog and human health used to develop an operational risk assessment tool for evaluating *Leishmania infantum* introduction and establishment in the United States through dog importation.

The consequence assessment considers biologic (health) consequences for further establishment of *L. infantum* in the general dog population and its impact on human and dog health (*19*). Health consequences are expected to vary depending on immune status, treatment response of infected humans and dogs, and the broader public health impact of autochthonous infections. Immunosuppressed persons are at greatest risk for major adverse consequences (Appendix reference *125*). Although uncommon, veterinary and research personnel may be infected while working with infected animals or biologic samples (Appendix references *126*). Other biologic considerations include illness and death, transmissibility, adverse treatment responses, and prognosis. We summarized consequence assessments for selected scenarios of *L. infantum* introduction through dog importation into the United States (Figure 9), which we adapted from the World Organisation for Animal Health (*19*).

Most *L. infantum* parasitic infections in dogs (85%–90%) and humans (95%–97%) are asymptomatic (*33*; Appendix references *127,128*). Reports suggest that <25% of exposed dogs in endemic areas mount effective immune responses and might resolve infection, even without treatment (Appendix references *129,130*). Asymptomatic infected dogs on appropriate SFPs are unlikely to create major public health risks for immunocompetent persons nearby (*29*,*33*).

CanL is rarely diagnosed in the United States, and veterinary professionals’ unfamiliarity could lead to delayed clinical suspicion and diagnosis. Once diagnosed, prognosis depends on the dog’s clinical stage and renal function when therapy is initiated (Appendix reference *130*). Among symptomatic nonproteinuric dogs treated for CanL, 75% survive >4 years, but in dogs with proteinuria, an indicator of impaired renal function, mean survival time is reduced to ≈2 years (Appendix references *130,131*). Although dogs never reach parasitic cure, clinical signs improve rapidly after therapy, and prognosis is favorable with frequent veterinary monitoring (Appendix references *104,113,130,132*). Treated dogs are much less infectious for months after treatment, reducing public health risks (*33*; Appendix references *104,133*).

Many medical professionals are unaware of risk factors and clinical signs suggestive of ZVL (*15*), and underreporting is likely extensive, even in areas where the disease is endemic (Appendix reference *134*). Delayed diagnosis and therapy can negatively impact health outcomes and increase vectorborne transmission in vulnerable populations (*15*; Appendix references *135,136*). Asymptomatic humans can transmit *L. infantum* parasites via blood transfusions, organ transplants, and sand fly bites but to a lesser extent than for immunocompromised or symptomatic persons (Appendix references *137,138*).

ZVL in humans is life-threatening if not appropriately treated. ZVL is especially problematic in vulnerable populations and can persist for decades after treatment (*8*). Persons concurrently infected with HIV generally have suboptimal clinical responses to leishmania treatment and are more likely to suffer posttreatment infection relapses (*12*; Appendix reference *125*). Treated immunocompetent humans often attain nonsterile cure and uncommonly develop disease after infection (*29*,*33*; Appendix reference *125*).

The US Food and Drug Administration (FDA) approved 2 medications for treating ZVL in humans, intravenous liposomal amphotericin B and oral miltefosine; no approved therapies are available for treating CanL in dogs. Besides allopurinol, access to leishmania treatment drugs for extralabel veterinary use is limited because of financial costs and availability within the United States.

Beyond biologic considerations, other factors need to be considered in a consequence assessment. Environmental consequences include potential *L. infantum* establishment in wildlife reservoirs and vectors, and environmental degradation from residual insecticides. Economic considerations include surveillance, diagnostic methods, treatment, and monitoring of dog and human cases, and preventative costs for SFPs on infected and susceptible dogs. Quantitative analysis of economic and environmental consequences of *L. infantum* infection is beyond the scope of this risk assessment and future research could elaborate on the impacts of establishment and spread of *L. infantum* parasites in the United States. However, expenses would result from detection of additional human and animal cases, treatment for infected persons, and vector control to prevent further spread. Because no licensed vaccine is approved in the United States, control efforts would primarily focus on treating infectious dogs and enforcing strict lifelong use of dog SFPs (Appendix references *139,140*).

### Consequence Assessment Uncertainty Level—Low

Sufficient studies characterize health consequences of establishment of autochthonous vectorborne transmission of ZVL and CanL in the United States. *L. infantum* parasites infect cats, rodents, and numerous other animals, but the impact of those animals on disease transmission in endemic areas is unknown (Appendix references *119,141–143*). Other natural *L. infantum* reservoirs and their role in transmission cycles need investigation (Appendix references *137,142,144*).

### Final Risk Estimation Matrix

**Figure 10:**
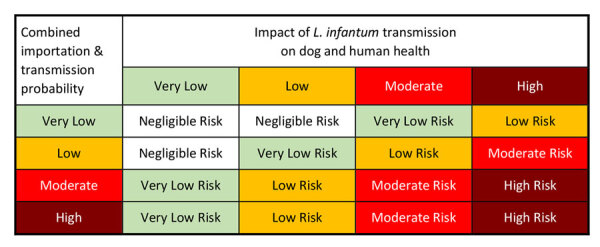
Final risk estimation matrix from an operational risk assessment tool for evaluating *Leishmania infantum* introduction and establishment in the United States through dog importation. Modified from Biosecurity Import Risk Analysis Guidelines (*21*).

A final risk estimation matrix (Figure 10) combines the probabilities of importation and vectorborne transmission (Figure 8) with the outputs of the consequence assessment. The resulting matrix enables risk assessors to evaluate the overall risk for introduction and establishment of *L. infantum* parasites in the United States through dog importation. Once the overall risk is determined, stakeholders can devise and employ strategies to mitigate the resulting risk.

### Risk Management

Risk management strategies include those that prevent *L. infantum* transmission by sand fly bites, between dogs, and from dogs to humans. SFPs should be applied throughout periods of sand fly activity to prevent sand fly bites. Both topical formulations containing permethrin, imidacloprid, indoxacarb, fipronil, or combinations of those drugs, and collars impregnated with deltamethrin or flumethrin/imidacloprid combination prevent sand fly bites and are available in the United States (Appendix reference *70*). Topical formulations require reapplication every 3–4 weeks and should be applied >2 days before potential exposure (*6*). Insecticide-impregnated collars can protect for 8–12 months and should be applied >1–2 weeks before potential exposure (*6*; Appendix reference *70*). Infected dogs residing in or traveling to areas in the United States with potential permissible sand fly vectors should have appropriate SFPs applied according to product label directions, regardless of treatment status (Appendix reference *115*). In some areas, that process might mean continual, year-round SFP use.

Dogs potentially infected with *L. infantum* should be excluded from breeding and blood donation activities until tested by quantitative serology 6 months after arrival in the United States and, if positive, permanently restricted from those activities (*6*; Appendix reference *145*). Because horizontal transmission risks between dogs are possible (*26*,*27*; Appendix references *146,147*), contact between potentially infected dogs and healthy seronegative dogs should be limited until quantitative serology results are available.

Vaccination for leishmaniosis can interfere with serologic testing (Appendix reference *148*), but vaccination status should not preclude SFP use or testing and subsequent exclusion of seropositive dogs from breeding or blood donation. Needlesticks and open wound contamination could result in zoonotic *L. infantum* transmission to humans. Veterinary staff should wear gloves and exercise caution when handling infected animals to prevent accidental exposures. Immunocompromised persons should take extra precautions when handling infected animals, for example by covering open wounds and washing hands immediately after contact. Persons with zoonotic disease exposures should consult their healthcare providers. Providers can consult CDC’s Parasitic Diseases Branch (404-718-4745 or parasites@cdc.gov).

### Canine Leishmaniosis ORAT

The ORAT lays out the 5-step risk assessment process for importing potentially infected dogs from *L. infantum­–*endemic countries (Appendix). First, assessors should determine the probability of importing an infected dog by considering the dog’s country of origin, how long the dog was in the country of origin and its lifestyle there, whether the dog was maintained on SFPs, and the results of diagnostic testing. Second, assessors should determine the probability of vectorborne transmission in the region of the United States where the dog is going, and whether sand flies are present and are competent vectors. Third, assessors should determine the combined probability of events from steps 1 and 2, using the combined probability table. Fourth, assessors should determine the impact on individual canine and human health, considering both horizontal and vertical transmission among dogs as well as iatrogenic and vectorborne transmission in both dogs and humans. Finally, assessors should determine the final risk estimate using the combined risk estimate table. We provide case studies that demonstrate the application of the ORAT and examples of appropriate risk mitigation strategies (Appendix).

## Conclusions

*L. infantum* parasites are a major hazard in dogs imported from endemic areas into the United States. Risks vary depending on factors specific to the dog and to geographic and human factors. Dog-specific factors include prevalence of leishmaniosis in the originating country, in-country SFP use to prevent sand fly bites, serologic and clinical status, and the dog’s occupation or lifestyle. Geographic factors include presence of the potentially permissive sand fly vector species, *Lu. shannoni*. Human factors include owner compliance with recommendations regarding SFP use and excluding seropositive dogs from breeding. US domestic *L. infantum* transmission would incur substantial healthcare costs, surveillance, and control efforts. The *L. infantum* ORAT provides public health and animal health officials with a framework to comprehensively assess risks posed by imported dogs and recommends mitigation measures to prevent endemic *L. infantum* transmission and establishment in the United States.

Appendix Additional information on an operational risk assessment tool for evaluating *Leishmania infantum* introduction and establishment in the United States through dog importation.
