# Supplementary material for: Operational Risk Assessment Tool for Evaluating Leishmania infantum Introduction and Establishment in the United States through Dog Importation
Source: Emerg Infect Dis. 2024 Dec;30(12):e231084. doi: 10.3201/eid3012.231084 (PMC11616669; doi:10.3201/eid3012.231084)
Supplement: Appendix — Additional information on an operational risk assessment tool for evaluating Leishmania infantum introduction and establishment in the United States through dog importation. [file 23-1084-Techapp-s1.pdf]

Article DOI: https://doi.org/10.3201/eid3012.231084

EID cannot ensure accessibility for supplementary materials supplied by authors. Readers who have difficulty accessing supplementary content should contact the authors for assistance.

Operational Risk Assessment Tool for Evaluating *Leishmania infantum* Introduction and Establishment in the United States through Dog Importation

Appendix

Operational Risk Assessment Tool

Step 1. Entry assessment: The entry assessment describes biologic pathways needed for US importation of a *L. infantum*-infected dog and estimates the probability of occurrence. Probability is impacted by imported country of origin, sand fly preventative (SFP) use during travel, duration of time spent in endemic country, dog occupation and outdoor exposure to sand flies, clinical progression of infection; *L. infantum*–infected dams (Appendix Figure 1).

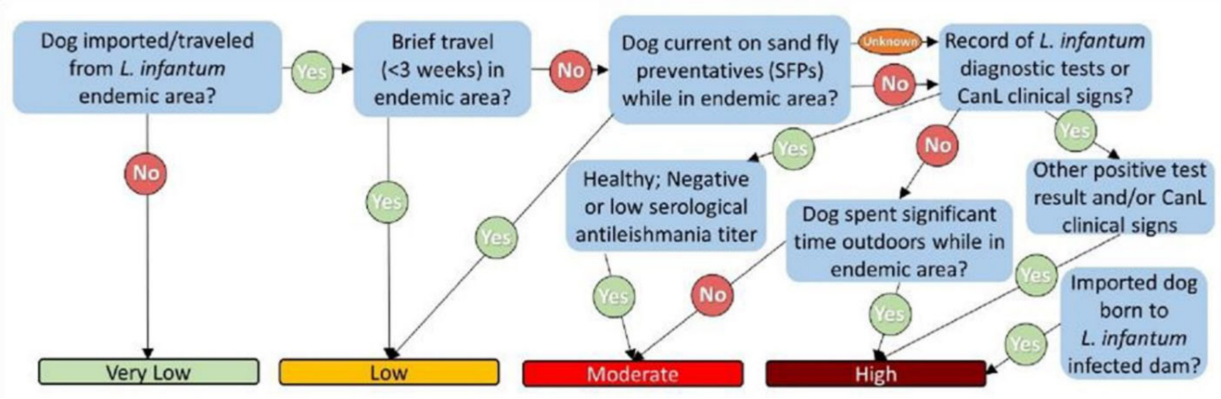

Appendix Figure 1. Schematic for determining probability of importing a *Leishmania infantum*–infected dog.

Step 2. Exposure assessment: The exposure assessment describes biologic pathways needed for transmission of *L. infantum* from an infected imported dog and subsequent exposure of humans and other animals in the US, and estimates the probability of occurrence. Probability

is impacted by sand fly preventative (SFP) use during infection, presence of permissible sand fly species, leishmania treatment use during infection, dog occupation and outdoor exposure to sand flies, and weather conditions that affect vector competence (Appendix Figure 2).

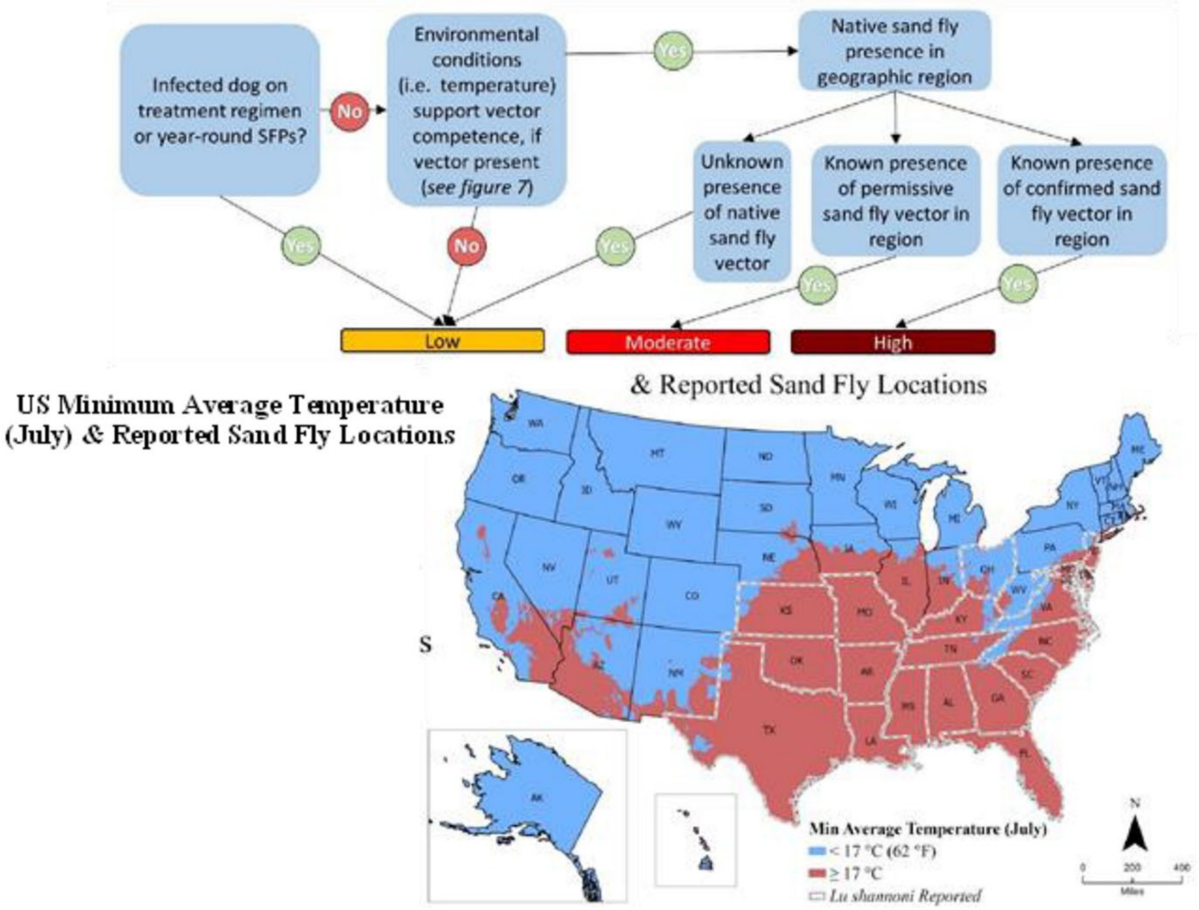

Appendix Figure 2. Schematic for determining probability of vectorborne transmission. Blue areas indicate average temperatures are less likely to support vector competence; red areas indicate average temperatures are more likely to support vector competence; white outline shows states with reported permissive vector species, *Lu. shannoni*.

Step 3. Determine combined probability of events: use the combination probability matrix below to determine conditional probability estimates of *L. infantum* importation via infected dogs followed by vector borne transmission in the US via sand flies (Appendix Figure 3).

Vectored transmission via sand flies	Importation of *L. infantum*-infected dogs			
	Very Low	Low	Moderate	High
Low	Very Low	Low	Low	Low
Moderate	Low	Low	Moderate	Moderate
High	Low	Moderate	Moderate	High

Appendix Figure 3. Combination probability matrix for determining conditional probability estimates of *L. infantum* importation via infected dogs after vectorborne transmission via sand flies in the United States.

Step 4. Consequence assessment: The consequence assessment describes the impact on human and dog health if *L. infantum* is established in the general US dog population (Appendix Figure 4). After considering the likelihood of potential scenarios, the impact can be estimated.

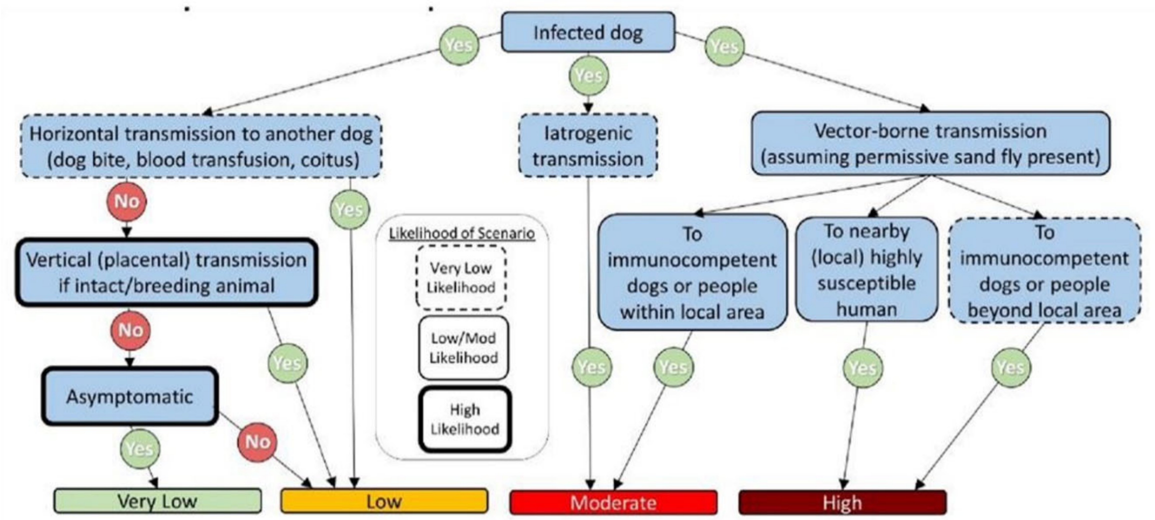

Appendix Figure 4. Consequence assessment for effects on human and dog health if *L. infantum* is established in the general U.S. dog population.

Step 5. Estimate final risk: use the final risk estimation matrix below to determine the *L. infantum* importation risk in dogs from endemic countries based on likelihood of importation and vector borne transmission and its consequences on human and dog health (Appendix Figure 5).

Combined importation & transmission probability	Impact of *L. infantum* transmission on dog and human health			
	Very Low	Low	Moderate	High
Very Low	Negligible Risk	Negligible Risk	Very Low Risk	Low Risk
Low	Negligible Risk	Very Low Risk	Low Risk	Moderate Risk
Moderate	Very Low Risk	Low Risk	Moderate Risk	High Risk
High	Very Low Risk	Low Risk	Moderate Risk	High Risk

Appendix Figure 5. Risk estimation matrix or determining the *L. infantum* importation risk in dogs from endemic countries based on likelihood of importation and vectorborne transmission and its consequences on human and dog health.

A complete reference guide to the risk assessment tool is provided on the last 2 pages of this appendix.

Case Study Examples

Case 1

A 10-year-old intact female spaniel dog that was adopted in Spain is moving to Georgia. The dog's current veterinarian reports that there are no open skin lesions, but the dog did have a mass on the leg that was biopsied and revealed *Leishmania*. The dog was tested via quantitative serology and is seropositive at a titer 2-fold higher than the laboratory's established cutoff. The dog is intended to be used as a breeding animal and has not been maintained on sand fly preventatives (SFPs).

Risk Assessment

Step 1 – Determine probability of importing an infected dog: high. This dog originated from an endemic country, is symptomatic for leishmaniosis, and the diagnosis was confirmed by serology and histopathology.

Step 2 – Determine probability of vector-borne transmission in the US: moderate. *Lutzomyia shannoni*, a suspected but unconfirmed vector of *Leishmania infantum* is present in Georgia.

This dog has not previously been maintained on sand fly preventatives.

Step 3 – Determine combined probability of events: moderate. The importation probability for this dog is high while the probability of vector-borne transmission in the US is moderate, thus the combined probability of events using the table in step 3 is moderate (Appendix Figure 6).

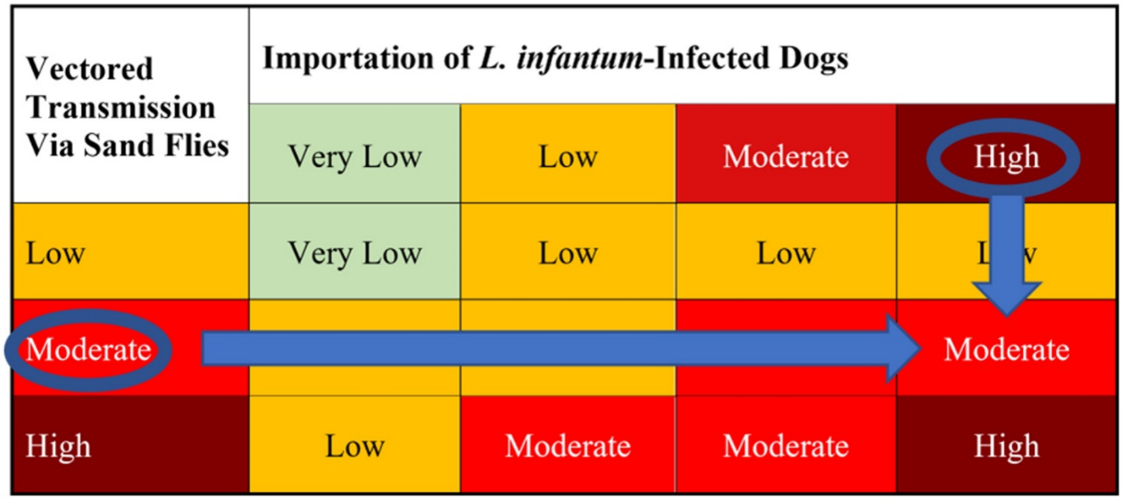

Appendix Figure 6. Combined probability matrix for release and exposure assessments for case 1.

Step 4 – Determine the impact on individual canine/human health (Appendix Figure 7).

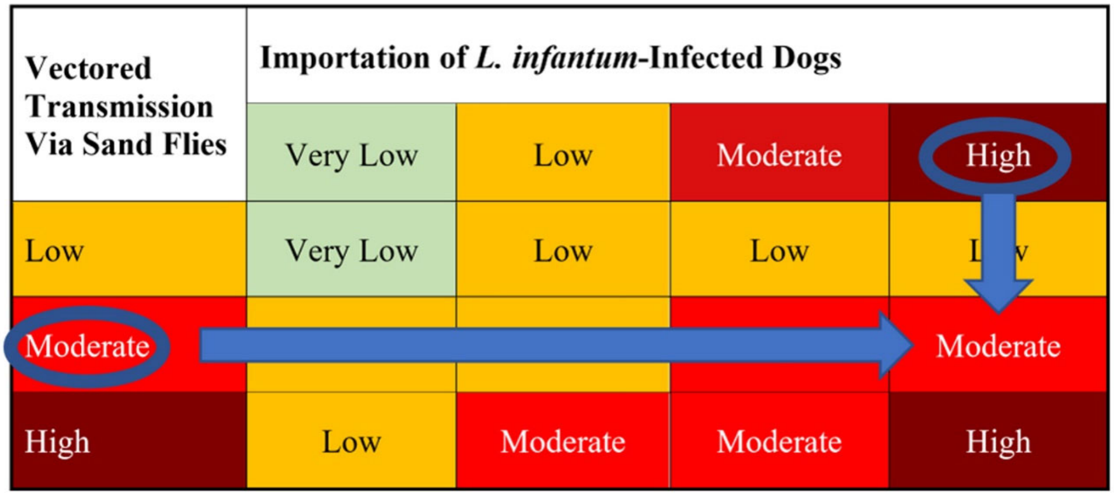

Appendix Figure 7. Final risk estimation matrix for evaluating the introduction and establishment of *L. infantum* in the United States through dog importation case 1.

• Step 4a: Horizontal/vertical transmission: This dog was intended to be used as a breeding animal, so the probability of horizontal and vertical transmission is high (the dark circle around vertical transmission on the left side of the diagram) and the impact is low.

• Step 4b: Zoonotic transmission: The dog is symptomatic and confirmed infected, but the probability of direct zoonotic transmission is considered very low (the dotted line circle around zoonotic transmission in the middle arm of the diagram), however the owner is immunocompromised. The impact is considered moderate.

• Step 4c: Vector-borne transmission: The probability of vector-borne transmission is moderate but given that the owner is immunocompromised the impact to the nearby susceptible human population is considered high.

• Step 4d: Select the greatest impact from steps 4a-4c for inclusion in step 5. The greatest of the potential impacts on canine and human health was high (from step 4c).

Step 5 – Determine the final risk estimate. From step 3 the combined probability of events was moderate. Taking all potential event probabilities and potential impacts together, the impact to canine and human health would be considered high (Appendix Figure 8). From the table in step 5, the final risk estimate is high.

Combined Importation & Transmission Probability	Impact of autochthonous transmission of *L. infantum* on dog and human health			
	Very Low	Low	Moderate	H[illegible]gh
Very Low	**Negligible Risk**	**Negligible Risk**	**Very Low Risk**	**Low R[illegible]k**
Low	**Negligible Risk**	**Very Low Risk**	**Low Risk**	**Mo[illegible]rate R[illegible]k**
[illegible]oderate	**[illegible] Risk**	[illegible]	**[illegible] Risk**	**High Risk**
High	**Very Low Risk**	**Low Risk**	**Moderate Risk**	**High Risk**

Appendix Figure 8. Final estimation matrix for evaluating the introduction and establishment of *L. infantum* in the United States through dog importation from case 1.

Mitigation Strategies and Public Health Response

Always check and comply with any state/local regulations regarding the disposition of dogs with leishmaniosis. In the absence of state/local regulations that would prohibit the dog from entry into the state, animal/public health officials should consider contacting both the owner and the receiving veterinarian.

• Owner – counsel on appropriate prevention methods, e.g., reduce time spent outside during peak sandfly activity and ensure the dog is being maintained on appropriate SFPs to prevent sand fly bites. If authority exists, consider requiring the animal to be sterilized; otherwise advise owner to prohibit animal from breeding. Counsel owner on potential for horizontal transmission to other dogs and zoonotic transmission to people. Because the owner is immunocompromised, they should take extra precautions when handling the dog (e.g., avoiding contact with open wounds and washing hands immediately after handling the dog).

• Veterinarian – determine if treatment is a possibility (as appropriate treatment may reduce a dog’s infectiousness); however, treatment is not a requirement as it rarely provides cure

and infected dogs can be managed safely otherwise. Discuss importance of appropriate SFP use and education for owner. Ensure veterinary staff are aware of possibility of iatrogenic transmission through needlesticks and take appropriate precautions. Ensure dog is not used for blood donation.

Case 2

A rescue organization located in Seattle, Washington wants to import a 3 year old intact male shepherd dog from Turkey. The dog was a stray, has no known clinical history, but is reportedly healthy. The dog was tested for leishmaniosis in Turkey by quantitative serology and the results are positive at the cutoff value established by the laboratory. The rescue organization requires that dogs be sterilized before placing them in homes and only adopts to people living in Washington and Oregon.

Risk Assessment

Step 1 – Determine probability of importing an infected dog: high. This dog originated from an endemic country, has unknown clinical history, and unknown use of sand fly preventatives. Testing for leishmaniosis indicates the dog may be infected, but the titer is low.

Step 2 – Determine probability of vector-borne transmission in the US: low. Although the dog has likely not been maintained on appropriate sand fly preventatives, suspected permissive sand fly vectors are not present in Washington or Oregon (where the dog might be placed).

Step 3 – Determine combined probability of events: low. The importation probability for this dog is high while the probability of vector-borne transmission in the US is low, thus the combined probability of events using the table in step 3 is low (Appendix Figure 9).

Vectored Transmission Via Sand Flies	Importation of *L. infantum*-Infected Dogs			
	Very Low	Low	Moderate	High
Low	[illegible]	[illegible]	[illegible]	Low
Moderate	Low	Low	Moderate	Moderate
High	Low	Moderate	Moderate	High

Appendix Figure 9. Combined probability matrix for release and exposure assessments from case 2.

Step 4 – Determine the impact on individual canine/human health.

• Step 4a: Horizontal/vertical transmission: The probability of horizontal transmission is low but is dependent on whether the dog will be placed with other dogs. The probability of vertical transmission is negligible since the dog will be sterilized before being rehomed. The dog is also asymptomatic. The overall impact in this scenario is considered very low (if not housed with other dogs) to low.

• Step 4b: Zoonotic transmission: The dog is asymptomatic but likely infected based on quantitative serologic results. The probability of direct zoonotic transmission is considered very low (the dotted line circle around zoonotic transmission in the middle arm of the diagram). The impact is considered moderate.

• Step 4c: Vector-borne transmission: The probability of vector-borne transmission is low as potentially permissive sand fly vectors are not present in the area.

• Step 4d: Select the greatest impact from steps 4a-4c for inclusion in step 5. The greatest of the potential impacts on canine and human health was moderate (from step 4b).

Step 5 – Determine the final risk estimate. From step 3 the combined probability of events was low. Taking all potential event probabilities and potential impacts together, (step 4) the impact to canine and human health would be considered moderate. From the table in step 5, the final risk estimate is low (Appendix Figure 10).

Combined Importation & Transmission Probability	Impact of autochthonous transmission of *L. infantum* on dog and human health			
	Very Low	Low	Moderate	High
Very Low	Negligible Risk	Negligible Risk	Ver[illegible]ow R[illegible]k	Low Risk
Low	[illegible] Risk	[illegible] Risk	Low Risk	Moderate Risk
Moderate	Very Low Risk	Low Risk	Moderate Risk	High Risk
High	Very Low Risk	Low Risk	Moderate Risk	High Risk

Appendix Figure 10. Final risk estimation matrix for evaluating the introduction and establishment of *L. infantum* in the United States through dog importation from case 2.

Mitigation Strategies and Public Health Response

Always check and comply with any state/local regulations regarding the disposition of dogs with leishmaniosis. In this scenario, the rescue organization should be informed of the overall risk assessment and consider it during the adoption process. Potential adoptees of this dog should be informed of the risk assessment results and educated about leishmaniosis; adoptees with immunocompromised family members or who may come into contact with immunocompromised persons should be informed of the potential risks of zoonotic transmission and advised accordingly. Recommend recheck exam and serology in 6 months and consider sand fly preventives.

Appendix References

51. Mattin M, Brodbelt D, Wylie C, Carbonell Antoñanzas M, Solano Gallego L, Espejo L, et al. Data collection to characterise the impact of canine leishmaniosis and modelling of the role of animals in spreading Leishmania infantum within the European Union. EFSA Supporting Publications. 2013;11:EN-466. https://doi.org/10.2903/sp.efsa.2014.EN-466

52. Calzetta L, Pistocchini E, Ritondo BL, Roncada P, Palma E, di Cave D, et al. Immunoprophylaxis pharmacotherapy against canine leishmaniosis: a systematic review and meta-analysis on the efficacy of vaccines approved in European Union. Vaccine. 2020;38:6695–703. PubMed https://doi.org/10.1016/j.vaccine.2020.08.051

53. Troncarelli MZ, Camargo JB, Machado JG, Lucheis SB, Langoni H. *Leishmania* spp. and/or *Trypanosoma cruzi* diagnosis in dogs from endemic and nonendemic areas for canine visceral leishmaniasis. Vet Parasitol. 2009;164:118–23. PubMed https://doi.org/10.1016/j.vetpar.2009.06.027

54. World Organisation for Animal Health. Manual of diagnostic tests and vaccines for terrestrial animals: 3.1.11. Leishmaniosis. 2022 Jan 12 [cited 22 May 23] https://www.woah.org/fileadmin/Home/eng/Health_standards/tahm/3.01.11_LEISHMANIOSIS.pdf

55. Solano-Gallego L, Morell P, Arboix M, Alberola J, Ferrer L. Prevalence of *Leishmania infantum* infection in dogs living in an area of canine leishmaniasis endemicity using PCR on several tissues and serology. J Clin Microbiol. 2001;39:560–3. PubMed https://doi.org/10.1128/JCM.39.2.560-563.2001

56. Gálvez R, Montoya A, Fontal F, Martínez De Murguía L, Miró G. Controlling phlebotomine sand flies to prevent canine *Leishmania infantum* infection: a case of knowing your enemy. Res Vet Sci. 2018;121:94–103. PubMed https://doi.org/10.1016/j.rvsc.2018.10.008

57. Otranto D, Paradies P, Lia RP, Latrofa MS, Testini G, Cantacessi C, et al. Efficacy of a combination of 10% imidacloprid/50% permethrin for the prevention of leishmaniasis in kennelled dogs in an endemic area. Vet Parasitol. 2007;144:270–8. PubMed https://doi.org/10.1016/j.vetpar.2006.09.012

58. Gavgani AS, Hodjati MH, Mohite H, Davies CR. Effect of insecticide-impregnated dog collars on incidence of zoonotic visceral leishmaniasis in Iranian children: a matched-cluster randomised trial. Lancet. 2002;360:374–9. PubMed https://doi.org/10.1016/S0140-6736(02)09609-5

59. Wylie CE, Carbonell-Antoñanzas M, Aiassa E, Dhollander S, Zagmutt FJ, Brodbelt DC, et al. A systematic review of the efficacy of prophylactic control measures for naturally occurring canine leishmaniosis. Part II: topically applied insecticide treatments and prophylactic medications. Prev Vet Med. 2014;117:19–27. PubMed https://doi.org/10.1016/j.prevetmed.2014.06.016

60. Silva SCPFE, Gomes LB, Carvalho PCFB, Santos AGRC, Borges LFNM, Oliveira CSF, et al. Effectiveness of the mass use of deltamethrin-impregnated dog collars for preventing transmission of canine leishmaniasis by *Lutzomyia* spp.: a cluster randomized controlled trial. Prev Vet Med. 2019;171:104770. PubMed https://doi.org/10.1016/j.prevetmed.2019.104770

61. Ligda P, Gizzarelli M, Kostopoulou D, Foglia Manzillo V, Saratsis A, Saratsi K, et al. Determination of the effect of collars containing 10% w/w imidacloprid and 4.5% w/w flumethrin (Seresto®) on the incidence of *Leishmania* and other canine vector-borne pathogen infections in Greece. Parasit Vectors. 2023;16:89. PubMed https://doi.org/10.1186/s13071-023-05678-4

62. Evans A, Bongiorno G, Fourie JJ, Lekouch N, Bianchi R, Khoury C, et al. Elevated and sustained anti-feeding effect of Scalibor® deltamethrin collar against the sand fly *Phlebotomus perniciosus* in dogs confirmed for 1 year following treatment. Med Vet Entomol. 2022;36:14–9. PubMed https://doi.org/10.1111/mve.12545

63. Yimam Y, Mohebali M. Effectiveness of insecticide-impregnated dog collars in reducing incidence rate of canine visceral leishmaniasis: a systematic review and meta-analysis. PLoS One. 2020;15:e0238601. PubMed https://doi.org/10.1371/journal.pone.0238601

64. Queiroga TBD, Ferreira HRP, Dos Santos WV, de Assis ABL, de Araújo Neto VT, da Câmara ACJ, et al. Fluralaner (Bravecto®) induces long-term mortality of *Lutzomyia longipalpis* after a blood meal in treated dogs. Parasit Vectors. 2020;13:609. PubMed https://doi.org/10.1186/s13071-020-04489-1

65. Apostolopoulos N, Mitropoulou A, Thom N, Moritz A. Update on therapy and prevention of canine leishmaniasis [in German]. Tierarztl Prax Ausg K Kleintiere Heimtiere. 2018;46:315–22. PubMed

66. Gomez SA, Curdi JL, Hernandez JAC, Peris PP, Gil AE, Velasquez RVO, et al. Phlebotomine mortality effect of systemic insecticides administered to dogs. Parasit Vectors. 2018;11:230. PubMed https://doi.org/10.1186/s13071-018-2820-x

67. Gomez SA, Picado A. Systemic insecticides used in dogs: potential candidates for phlebotomine vector control? Trop Med Int Health. 2017;22:755–64. PubMed https://doi.org/10.1111/tmi.12870

68. Pugliese M, Gaglio G, Passantino A, Brianti E, Napoli E. Natural products against sand fly vectors of leishmaniosis: a systematic review. Vet Sci. 2021;8:150. PubMed https://doi.org/10.3390/vetsci8080150

69. Zatelli A, Fondati A, Maroli M; Canine Leishmaniosis Working Group. The knowns and unknowns of the efficacy of neem oil (*Azadirachta indica*) used as a preventative measure against *Leishmania* sand fly vectors (*Phlebotomus* genus). Prev Vet Med. 2022;202:105618. PubMed https://doi.org/10.1016/j.prevetmed.2022.105618

70. Miró G, Petersen C, Cardoso L, Bourdeau P, Baneth G, Solano-Gallego L, et al. Novel areas for prevention and control of canine leishmaniosis. Trends Parasitol. 2017;33:718–30. PubMed https://doi.org/10.1016/j.pt.2017.05.005

71. Meyers AC, Auckland L, Meyers HF, Rodriguez CA, Kontowicz E, Petersen CA, et al. Epidemiology of vector-borne pathogens among U.S. government working dogs. Vector Borne Zoonotic Dis. 2021;21:358–68. PubMed https://doi.org/10.1089/vbz.2020.2725

72. Otranto D, Dantas-Torres F, Mihalca AD, Traub RJ, Lappin M, Baneth G. Zoonotic parasites of sheltered and stray dogs in the era of the global economic and political crisis. Trends Parasitol. 2017;33:813–25. PubMed https://doi.org/10.1016/j.pt.2017.05.013

73. Miró G, Montoya A, Mateo M, Alonso A, García S, García A, et al. A leishmaniosis surveillance system among stray dogs in the region of Madrid: ten years of serodiagnosis (1996–2006). Parasitol Res. 2007;101:253–7. PubMed https://doi.org/10.1007/s00436-007-0497-8

74. Davoust B, Roqueplo C, Parzy D, Watier-Grillot S, Marié JL. A twenty-year follow-up of canine leishmaniosis in three military kennels in southeastern France. Parasit Vectors. 2013;6:323. PubMed https://doi.org/10.1186/1756-3305-6-323

75. Gálvez R, Miró G, Descalzo MA, Nieto J, Dado D, Martín O, et al. Emerging trends in the seroprevalence of canine leishmaniasis in the Madrid region (central Spain). Vet Parasitol. 2010;169:327–34. PubMed https://doi.org/10.1016/j.vetpar.2009.11.025

76. Hide M, Michel G, Legueult K, Pin R, Leonard S, Simon L, et al. Asymptomatic *Leishmania infantum* infection in dogs and dog owners in an endemic area in southeast France. Parasite. 2024;31:16. PubMed https://doi.org/10.1051/parasite/2024019

77. Killian JW. The impact of leishmaniasis on military working dogs with Mediterranean Basin exposure. US Army Med Dep J. 2007;6:17–25. PubMed

78. Bourdeau P, Saridomichelakis MN, Oliveira A, Oliva G, Kotnik T, Gálvez R, et al. Management of canine leishmaniosis in endemic SW European regions: a questionnaire-based multinational survey. Parasit Vectors. 2014;7:110. PubMed https://doi.org/10.1186/1756-3305-7-110

79. Adel A, Berkvens D, Abatih E, Soukehal A, Bianchini J, Saegerman C. Evaluation of immunofluorescence antibody test used for the diagnosis of canine leishmaniasis in the Mediterranean Basin: a systematic review and meta-analysis. PLoS One. 2016;11:e0161051. PubMed https://doi.org/10.1371/journal.pone.0161051

80. Otranto D, Dantas-Torres F. The prevention of canine leishmaniasis and its impact on public health. Trends Parasitol. 2013;29:339–45. PubMed https://doi.org/10.1016/j.pt.2013.05.003

81. Liénard E, Bouhsira E, Jacquiet P, Warin S, Kaltsatos V, Franc M. Efficacy of dinotefuran, permethrin and pyriproxyfen combination spot-on on dogs against *Phlebotomus perniciosus* and *Ctenocephalides canis*. Parasitol Res. 2013;112:3799–805. PubMed https://doi.org/10.1007/s00436-013-3568-z

82. Miró G, Gálvez R, Mateo M, Montoya A, Descalzo MA, Molina R. Evaluation of the efficacy of a topically administered combination of imidacloprid and permethrin against *Phlebotomus perniciosus* in dog. Vet Parasitol. 2007;143:375–9. PubMed https://doi.org/10.1016/j.vetpar.2006.09.014

83. Velez R, Gállego M. Commercially approved vaccines for canine leishmaniosis: a review of available data on their safety and efficacy. Trop Med Int Health. 2020;25:540–57. PubMed https://doi.org/10.1111/tmi.13382

84. Toepp A, Larson M, Wilson G, Grinnage-Pulley T, Bennett C, Leal-Lima A, et al. Randomized, controlled, double-blinded field trial to assess Leishmania vaccine effectiveness as immunotherapy for canine leishmaniosis. Vaccine. 2018;36:6433–41. PubMed https://doi.org/10.1016/j.vaccine.2018.08.087

85. Kamhawi S, Ramalho-Ortigao M, Pham VM, Kumar S, Lawyer PG, Turco SJ, et al. A role for insect galectins in parasite survival. Cell. 2004;119:329–41. PubMed https://doi.org/10.1016/j.cell.2004.10.009

86. Antoniou M, Gramiccia M, Molina R, Dvorak V, Volf P. The role of indigenous phlebotomine sandflies and mammals in the spreading of leishmaniasis agents in the Mediterranean region. Euro Surveill. 2013;18:20540. PubMed https://doi.org/10.2807/1560-7917.ES2013.18.30.20540

87. Volf P, Myskova J. Sand flies and *Leishmania*: specific versus permissive vectors. Trends Parasitol. 2007;23:91–2. PubMed https://doi.org/10.1016/j.pt.2006.12.010

88. Weng JL, Young SL, Gordon DM, Claborn D, Petersen C, Ramalho-Ortigao M. First report of phlebotomine sand flies (Diptera: Psychodidae) in Kansas and Missouri, and a PCR method to

distinguish *Lutzomyia shannoni* from *Lutzomyia vexator*. J Med Entomol. 2012;49:1460–5. PubMed https://doi.org/10.1603/ME12105

89. Price DC, Gunther DE, Gaugler R. First collection records of phlebotomine sand flies (Diptera: Psychodidae) from New Jersey. J Med Entomol. 2011;48:476–8. PubMed https://doi.org/10.1603/ME10170

90. Minter L, Kovacic B, Claborn DM, Lawyer P, Florin D, Brown GC. New state records for *Lutzomyia shannoni* and *Lutzomyia vexator*. J Med Entomol. 2009;46:965–8. PubMed https://doi.org/10.1603/033.046.0432

91. Claborn DM, Rowton ED, Lawyer PG, Brown GC, Keep LW. Species diversity and relative abundance of phlebotomine sand flies (Diptera: Psychodidae) on three Army installations in the southern United States and susceptibility of a domestic sand fly to infection with Old World *Leishmania major*. Mil Med. 2009;174:1203–8. PubMed https://doi.org/10.7205/MILMED-D-00-4309

92. Claborn D, Masuoka P, Morrow M, Keep L. Habitat analysis of North American sand flies near veterans returning from leishmania-endemic war zones. Int J Health Geogr. 2008;7:65. PubMed https://doi.org/10.1186/1476-072X-7-65

93. Kipp EJ, de Almeida M, Marcet PL, Bradbury RS, Benedict TK, Lin W, et al. An atypical case of autochthonous cutaneous leishmaniasis associated with naturally infected phlebotomine sand flies in Texas, United States. Am J Trop Med Hyg. 2020;103:1496–501. PubMed https://doi.org/10.4269/ajtmh.20-0107

94. Mann RS, Kaufman PE. The seasonal abundance of phlebotomine sand flies, *Lutzomyia* species in Florida. J Am Mosq Control Assoc. 2010;26:10–7. PubMed https://doi.org/10.2987/09-5901.1

95. Comer JA, Kavanaugh DM, Stallknecht DE, Ware GO, Corn JL, Nettles VF. Effect of forest type on the distribution of *Lutzomyia shannoni* (Diptera: Psychodidae) and vesicular stomatitis virus on Ossabaw Island, Georgia. J Med Entomol. 1993;30:555–60. PubMed https://doi.org/10.1093/jmedent/30.3.555

96. Solano-Gallego L, Koutinas A, Miró G, Cardoso L, Pennisi MG, Ferrer L, et al. Directions for the diagnosis, clinical staging, treatment and prevention of canine leishmaniosis. Vet Parasitol. 2009;165:1–18. PubMed https://doi.org/10.1016/j.vetpar.2009.05.022

97. Lewis DJ. Phlebotomid sandflies. Bull World Health Organ. 1971;44:535–51. PubMed

98. Hlavacova J, Votypka J, Volf P. The effect of temperature on *Leishmania* (Kinetoplastida: Trypanosomatidae) development in sand flies. J Med Entomol. 2013;50:955–8. PubMed https://doi.org/10.1603/ME13053

99. Fick SE, Hijmans RJ. WorldClim 2: new 1-km spatial resolution climate surfaces for global land areas. Int J Climatol. 2017;37:4302–15. https://doi.org/10.1002/joc.5086

100. Miró G, Gálvez R, Fraile C, Descalzo MA, Molina R. Infectivity to *Phlebotomus perniciosus* of dogs naturally parasitized with *Leishmania infantum* after different treatments. Parasit Vectors. 2011;4:52. PubMed https://doi.org/10.1186/1756-3305-4-52

101. Moreno J, Nieto J, Chamizo C, González F, Blanco F, Barker DC, et al. The immune response and PBMC subsets in canine visceral leishmaniasis before, and after, chemotherapy. Vet Immunol Immunopathol. 1999;71:181–95. PubMed https://doi.org/10.1016/S0165-2427(99)00096-3

102. Noli C, Saridomichelakis MN. An update on the diagnosis and treatment of canine leishmaniosis caused by *Leishmania infantum* (syn. *L. chagasi*). Vet J. 2014;202:425–35. PubMed https://doi.org/10.1016/j.tvjl.2014.09.002

103. Alvar J, Molina R, San Andrés M, Tesouro M, Nieto J, Vitutia M, et al. Canine leishmaniasis: clinical, parasitological and entomological follow-up after chemotherapy. Ann Trop Med Parasitol. 1994;88:371–8. PubMed https://doi.org/10.1080/00034983.1994.11812879

104. Gradoni L, Maroli M, Gramiccia M, Mancianti F. *Leishmania infantum* infection rates in *Phlebotomus perniciosus* fed on naturally infected dogs under antimonial treatment. Med Vet Entomol. 1987;1:339–42. PubMed https://doi.org/10.1111/j.1365-2915.1987.tb00364.x

105. Baneth G, Shaw SE. Chemotherapy of canine leishmaniosis. Vet Parasitol. 2002;106:315–24. PubMed https://doi.org/10.1016/S0304-4017(02)00115-2

106. Andrade HM, Toledo VP, Pinheiro MB, Guimarães TM, Oliveira NC, Castro JA, et al. Evaluation of miltefosine for the treatment of dogs naturally infected with *L. infantum* (=*L. chagasi*) in Brazil. Vet Parasitol. 2011;181:83–90. PubMed https://doi.org/10.1016/j.vetpar.2011.05.009

107. Gangneux JP, Dullin M, Sulahian A, Garin YJ, Derouin F. Experimental evaluation of second-line oral treatments of visceral leishmaniasis caused by *Leishmania infantum*. Antimicrob Agents Chemother. 1999;43:172–4. PubMed https://doi.org/10.1128/AAC.43.1.172

108. Noli C, Auxilia ST. Treatment of canine Old World visceral leishmaniasis: a systematic review. Vet Dermatol. 2005;16:213–32. PubMed https://doi.org/10.1111/j.1365-3164.2005.00460.x

109. Rougier S, Hasseine L, Delaunay P, Michel G, Marty P. One-year clinical and parasitological follow-up of dogs treated with marbofloxacin for canine leishmaniosis. Vet Parasitol. 2012;186:245–53. PubMed https://doi.org/10.1016/j.vetpar.2011.11.016

110. Bourdeau P, Rowton E, Petersen C. Impact of different *Leishmania* reservoirs on sand fly transmission: perspectives from xenodiagnosis and other one health observations. Vet Parasitol. 2020;287:109237. PubMed https://doi.org/10.1016/j.vetpar.2020.109237

111. Petersen CA. Leishmaniasis, an emerging disease found in companion animals in the United States. Top Companion Anim Med. 2009;24:182–8. PubMed https://doi.org/10.1053/j.tcam.2009.06.006

112. Maroli M, Gradoni L, Oliva G, Castagnaro M, Crotti A, Lubas G, et al. Guidelines for prevention of leishmaniasis in dogs. J Am Vet Med Assoc. 2010;236:1200–6. PubMed https://doi.org/10.2460/javma.236.11.1200

113. Solano-Gallego L, Miró G, Koutinas A, Cardoso L, Pennisi MG, Ferrer L, et al.; The LeishVet Group. LeishVet guidelines for the practical management of canine leishmaniosis. Parasit Vectors. 2011;4:86. PubMed https://doi.org/10.1186/1756-3305-4-86

114. Werneck GL, Figueiredo FB, Cruz MDSPE. Impact of 4% deltamethrin-impregnated dog collars on the incidence of human visceral leishmaniasis: a community intervention trial in Brazil. Pathogens. 2024;13:135. PubMed https://doi.org/10.3390/pathogens13020135

115. Gonçalves G, Campos MP, Gonçalves AS, Figueiredo FB. Therapeutic success and failure in using miltefosine to treat dogs naturally infected with *Leishmania infantum*. Rev Bras Parasitol Vet. 2024;33:e015023. PubMed https://doi.org/10.1590/s1984-29612024012

116. Yasur-Landau D, Jaffe CL, David L, Baneth G. Allopurinol resistance in *Leishmania infantum* from dogs with disease relapse. PLoS Negl Trop Dis. 2016;10:e0004341. PubMed https://doi.org/10.1371/journal.pntd.0004341

117. Aït-Oudhia K, Gazanion E, Sereno D, Oury B, Dedet JP, Pratlong F, et al. In vitro susceptibility to antimonials and amphotericin B of *Leishmania infantum* strains isolated from dogs in a region lacking drug selection pressure. Vet Parasitol. 2012;187:386–93. PubMed https://doi.org/10.1016/j.vetpar.2012.01.034

118. Miró G, Cardoso L, Pennisi MG, Oliva G, Baneth G. Canine leishmaniosis—new concepts and insights on an expanding zoonosis: part two. Trends Parasitol. 2008;24:371–7. PubMed https://doi.org/10.1016/j.pt.2008.05.003

119. Quinnell RJ, Courtenay O. Transmission, reservoir hosts and control of zoonotic visceral leishmaniasis. Parasitology. 2009;136:1915–34. PubMed https://doi.org/10.1017/S0031182009991156

120. Proverbio D, Spada E, Bagnagatti de Giorgi G, Perego R, Valena E. Relationship between *Leishmania* IFAT titer and clinicopathological manifestations (clinical score) in dogs. BioMed Res Int. 2014;2014:412808. PubMed https://doi.org/10.1155/2014/412808

121. Courtenay O, Quinnell RJ, Garcez LM, Shaw JJ, Dye C. Infectiousness in a cohort of Brazilian dogs: why culling fails to control visceral leishmaniasis in areas of high transmission. J Infect Dis. 2002;186:1314–20. PubMed https://doi.org/10.1086/344312

122. Travi BL, Tabares CJ, Cadena H, Ferro C, Osorio Y. Canine visceral leishmaniasis in Colombia: relationship between clinical and parasitologic status and infectivity for sand flies. Am J Trop Med Hyg. 2001;64:119–24. PubMed https://doi.org/10.4269/ajtmh.2001.64.119

123. Florin DALP, Lawyer P, Rowton E, Schultz G, Wilkerson R, Davies SJ, et al. Population dynamics of *Lutzomyia shannoni* (Diptera: Psychodidae) at the Patuxent National Wildlife Research Refuge, Maryland. J Am Mosq Control Assoc. 2010;26:337–9. PubMed https://doi.org/10.2987/10-6022.1

124. García-Castro A, Egui A, Thomas MC, López MC. Humoral and cellular immune response in asymptomatic dogs with visceral leishmaniasis: a review. Vaccines (Basel). 2022;10:947. PubMed https://doi.org/10.3390/vaccines10060947

125. Aronson N, Herwaldt BL, Libman M, Pearson R, Lopez-Velez R, Weina P, et al. Diagnosis and treatment of leishmaniasis: clinical practice guidelines by the Infectious Diseases Society of America (IDSA) and the American Society of Tropical Medicine and Hygiene (ASTMH). Clin Infect Dis. 2016;63:e202–64. PubMed https://doi.org/10.1093/cid/ciw670

126. Herwaldt B. Protozoa and helminths. In: Wooley DP, Byers KB, editors. Biological safety: principles and practices, 5th edition. Washington (DC): ASM Press; 2017. p. 105–45.

127. Otranto D, Paradies P, de Caprariis D, Stanneck D, Testini G, Grimm F, et al. Toward diagnosing *Leishmania infantum* infection in asymptomatic dogs in an area where leishmaniasis is endemic. Clin Vaccine Immunol. 2009;16:337–43. PubMed https://doi.org/10.1128/CVI.00268-08

128. Dantas-Torres F, de Brito ME, Brandão-Filho SP. Seroepidemiological survey on canine leishmaniasis among dogs from an urban area of Brazil. Vet Parasitol. 2006;140:54–60. PubMed https://doi.org/10.1016/j.vetpar.2006.03.008

129. Paradies P, Sasanelli M, de Caprariis D, Testini G, Traversa D, Lia RP, et al. Clinical and laboratory monitoring of dogs naturally infected by *Leishmania infantum*. Vet J. 2010;186:370–3. PubMed https://doi.org/10.1016/j.tvjl.2009.09.011

130. Roura X, Fondati A, Lubas G, Gradoni L, Maroli M, Oliva G, et al. Prognosis and monitoring of leishmaniasis in dogs: a working group report. Vet J. 2013;198:43–7. PubMed https://doi.org/10.1016/j.tvjl.2013.04.001

131. Geisweid K, Mueller R, Sauter-Louis C, Hartmann K. Prognostic analytes in dogs with *Leishmania infantum* infection living in a non-endemic area. Vet Rec. 2012;171:399. PubMed https://doi.org/10.1136/vr.100637

132. Slappendel RJ, Teske E. The effect of intravenous or subcutaneous administration of meglumine antimonate (Glucantime) in dogs with leishmaniasis. A randomized clinical trial. Vet Q. 1997;19:10–3. PubMed https://doi.org/10.1080/01652176.1997.9694729

133. Guarga JL, Moreno J, Lucientes J, Gracia MJ, Peribáñez MA, Alvar J, et al. Canine leishmaniasis transmission: higher infectivity amongst naturally infected dogs to sand flies is associated with lower proportions of T helper cells. Res Vet Sci. 2000;69:249–53. PubMed https://doi.org/10.1053/rvsc.2000.0419

134. Maia C, Conceição C, Pereira A, Rocha R, Ortuño M, Muñoz C, et al. The estimated distribution of autochthonous leishmaniasis by *Leishmania infantum* in Europe in 2005–2020. PLoS Negl Trop Dis. 2023;17:e0011497. PubMed https://doi.org/10.1371/journal.pntd.0011497

135. Medley GF, Hollingsworth TD, Olliaro PL, Adams ER. Health-seeking behaviour, diagnostics and transmission dynamics in the control of visceral leishmaniasis in the Indian subcontinent. Nature. 2015;528:S102–8. PubMed https://doi.org/10.1038/nature16042

136. McIlwee BE, Weis SE, Hosler GA. Incidence of endemic human cutaneous leishmaniasis in the United States. JAMA Dermatol. 2018;154:1032–9. PubMed https://doi.org/10.1001/jamadermatol.2018.2133

137. Costa CH, Stewart JM, Gomes RB, Garcez LM, Ramos PK, Bozza M, et al. Asymptomatic human carriers of *Leishmania chagasi*. Am J Trop Med Hyg. 2002;66:334–7. PubMed https://doi.org/10.4269/ajtmh.2002.66.334

138. Molina R, Jiménez M, García-Martínez J, San Martín JV, Carrillo E, Sánchez C, et al. Role of asymptomatic and symptomatic humans as reservoirs of visceral leishmaniasis in a Mediterranean

context. PLoS Negl Trop Dis. 2020;14:e0008253. PubMed https://doi.org/10.1371/journal.pntd.0008253

139. Vaz TP, Quaresma PF, Rêgo FD, Souza CB, Fontes G, Gontijo CMF. Clinical and laboratory response of domiciled dogs with visceral leishmaniasis treated with miltefosine and allopurinol. Trop Med Infect Dis. 2023;8:472. PubMed https://doi.org/10.3390/tropicalmed8100472

140. Gizzarelli M, Foglia Manzillo V, Inglese A, Montagnaro S, Oliva G. Retrospective long-term evaluation of miltefosine-allopurinol treatment in canine leishmaniosis. Pathogens. 2023;12:864. PubMed https://doi.org/10.3390/pathogens12070864

141. Martín-Sánchez J, Acedo C, Muñoz-Pérez M, Pesson B, Marchal O, Morillas-Márquez F. Infection by *Leishmania infantum* in cats: epidemiological study in Spain. Vet Parasitol. 2007;145:267–73. PubMed https://doi.org/10.1016/j.vetpar.2006.11.005

142. Molina R, Jiménez MI, Cruz I, Iriso A, Martín-Martín I, Sevillano O, et al. The hare (*Lepus granatensis*) as potential sylvatic reservoir of *Leishmania infantum* in Spain. Vet Parasitol. 2012;190:268–71. PubMed https://doi.org/10.1016/j.vetpar.2012.05.006

143. Travi BL, Osorio Y, Guarín N, Cadena H. *Leishmania* (*Leishmania*) *chagasi*: clinical and parasitological observations in experimentally infected *Didelphis marsupialis*, reservoir of New World visceral leishmaniasis. Exp Parasitol. 1998;88:73–5. PubMed https://doi.org/10.1006/expr.1998.4214

144. Aliaga L, Ceballos J, Sampedro A, Cobo F, López-Nevot MA, Merino-Espinosa G, et al. Asymptomatic *Leishmania* infection in blood donors from the Southern of Spain. Infection. 2019;47:739–47. PubMed https://doi.org/10.1007/s15010-019-01297-3

145. de Freitas E, Melo MN, da Costa-Val AP, Michalick MS. Transmission of *Leishmania infantum* via blood transfusion in dogs: potential for infection and importance of clinical factors. Vet Parasitol. 2006;137:159–67. PubMed https://doi.org/10.1016/j.vetpar.2005.12.011

146. Karkamo V, Kaistinen A, Näreaho A, Dillard K, Vainio-Siukola K, Vidgrén G, et al. The first report of autochthonous non-vector-borne transmission of canine leishmaniosis in the Nordic countries. Acta Vet Scand. 2014;56:84. PubMed https://doi.org/10.1186/s13028-014-0084-9

147. Daval N, Marchal C, Guillaumot L, Hüe T, Ravel C, Keck N, et al. First report of autochthonous non-vectorial canine leishmaniasis in New Caledonia, south-western Pacific: implications for new control measures and recommendations on importation of dogs. Parasit Vectors. 2016;9:108. PubMed https://doi.org/10.1186/s13071-016-1388-6

148. Solano-Gallego L, Cardoso L, Pennisi MG, Petersen C, Bourdeau P, Oliva G, et al. Diagnostic challenges in the era of canine *Leishmania infantum* vaccines. Trends Parasitol. 2017;33:706–17. PubMed https://doi.org/10.1016/j.pt.2017.06.004

149. World Health Organization. Leishmaniasis in high-burden countries: an epidemiological update based on data reported in 2014. Wkly Epidemiol Rec. 2016;91:287–96. PubMed

150. World Health Organization. Control of neglected tropical diseases (NTD). Geographical distribution of leishmaniasis clinical forms – zoonotic visceral leishmaniasis. Geneva: The Organization; 2019.

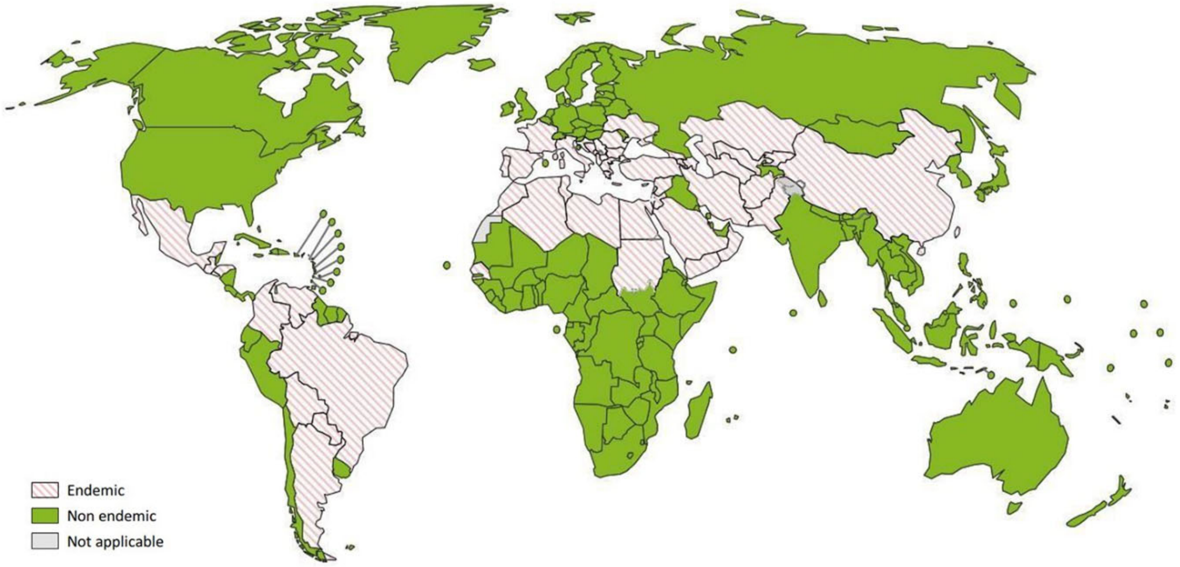

Appendix Figure 11. Map of *Leishmania infantum*–endemic countries. High burden country (*149*). Map and table reproduced from the World Health Organization WHO (*150*), by permission.

New World	
Argentina	Honduras
Bolivia	Mexico
**Brazil**†	Nicaragua
Columbia	**Paraguay**†
Costa Rica	Venezuela
El Salvador	
Guatemala	

Old World			
Afghanistan	**China**†	Greece	Kyrgyzstan
Albania	Croatia	Iran	Lebanon
Algeria	Cyprus	Iraq	Libyan Aral Jamahiriya
Armenia	Egypt	Israel	Macedonia
Azerbaijan	France	Italy	Malta
Bosnia and Herzegovina	Gambia	Jordan	Mauritania
Bulgaria	**Georgia**†	Kazakhstan	Monaco

Appendix Figure 12. Distribution of *Leishmania infantum* by country or territory, 2009. †High burden country (*149*). Map and table reproduced from the World Health Organization WHO (*150*), by permission.

Operational Risk Assessment Tool

Title: Evaluating *Leishmania infantum* risk in the United States through Dog Importation
Purpose: Provide a qualitative tool to assess risks of importing dogs with *L. infantum* into the US

Step 1. Entry assessment: The entry assessment describes biological pathways needed for US importation of a *L. infantum*-infected dog and estimates the probability of occurrence. Probability impacted by:

- Imported country of origin
- Duration of time spent in endemic country
- Clinical progression of infection
- Sand fly preventative (SFP) use during travel
- Dog occupation & outdoor exposure to sand flies
- *L. infantum*-infected dams

Determine the probability of importing an infected dog

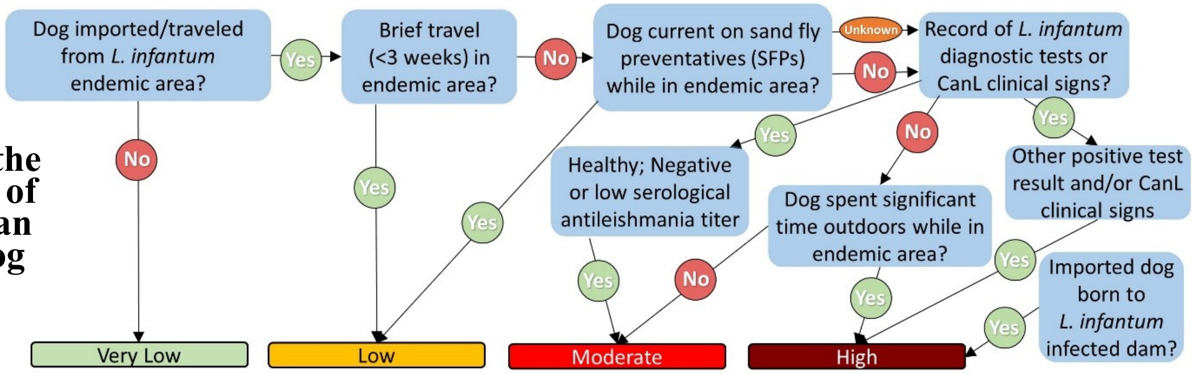

Step 2. Exposure assessment: The exposure assessment describes biological pathways needed for transmission of *L. infantum* from an infected imported dog and subsequent exposure of humans and other animals in the US, and estimates the probability of occurrence. Probability impacting by:

- Sand fly preventative (SFP) use during infection
- Anti-leishmanial treatment use during infection
- Weather conditions that affect vector competence
- Presence of permissible sand fly species
- Dog occupation & outdoor exposure to sand flies

Determine the probability of vector-borne transmission

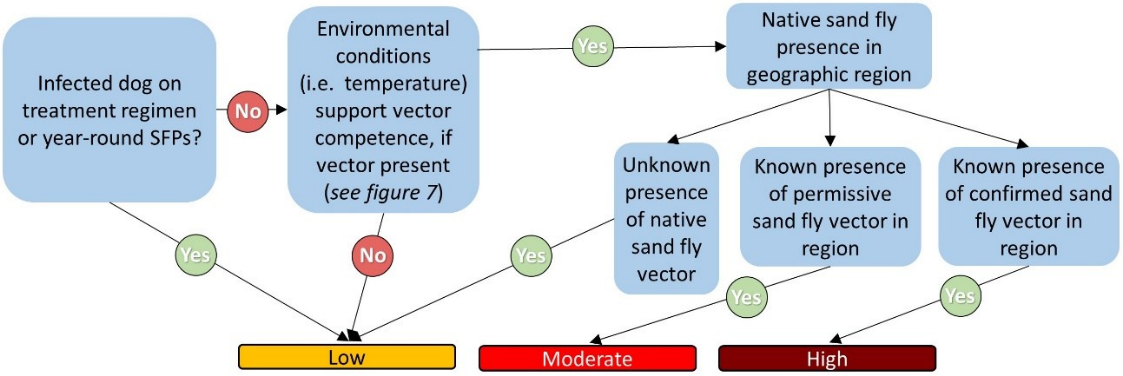

US Minimum Average Temperature (July) & Reported Sand Fly Locations

Blue areas —Average temps are less likely to support vector competence

Red areas—Average temps are more likely to support vector competence

White outline shows states with reported permissive vector (*Lu. shannoni*)

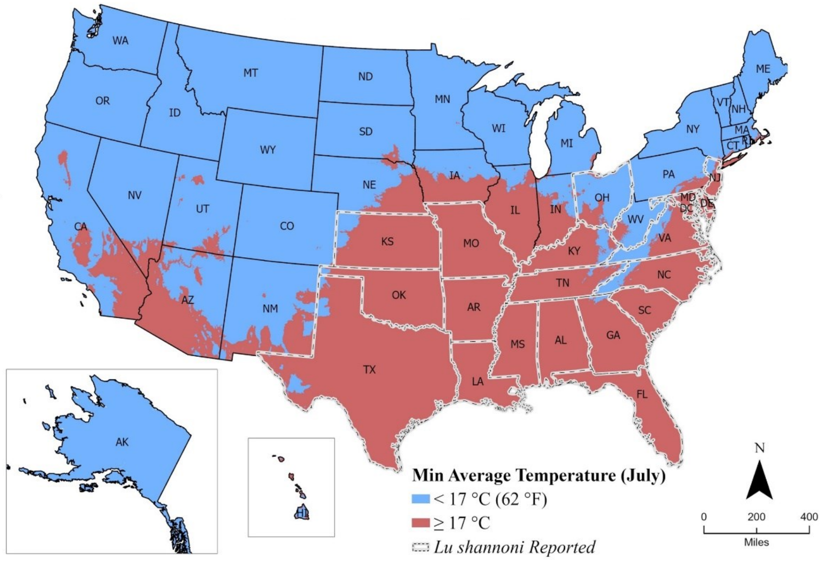

Step 3. Determine combined probability of events: use the combination probability matrix below to determine conditional probability estimates of *L. infantum* importation via infected dogs followed by vector borne transmission in the US via sand flies.

**Vectored transmission via sand flies**	**Importation of *L. infantum*-infected dogs**			
	Very Low	Low	Moderate	High
Low	Very Low	Low	Low	Low
Moderate	Low	Low	Moderate	Moderate
High	Low	Moderate	Moderate	High

Step 4. Consequence assessment: The consequence assessment describes the impact on human and dog health if *L. infantum* is established in the general US dog population. After considering the likelihood of potential scenarios, the impact can be estimated:

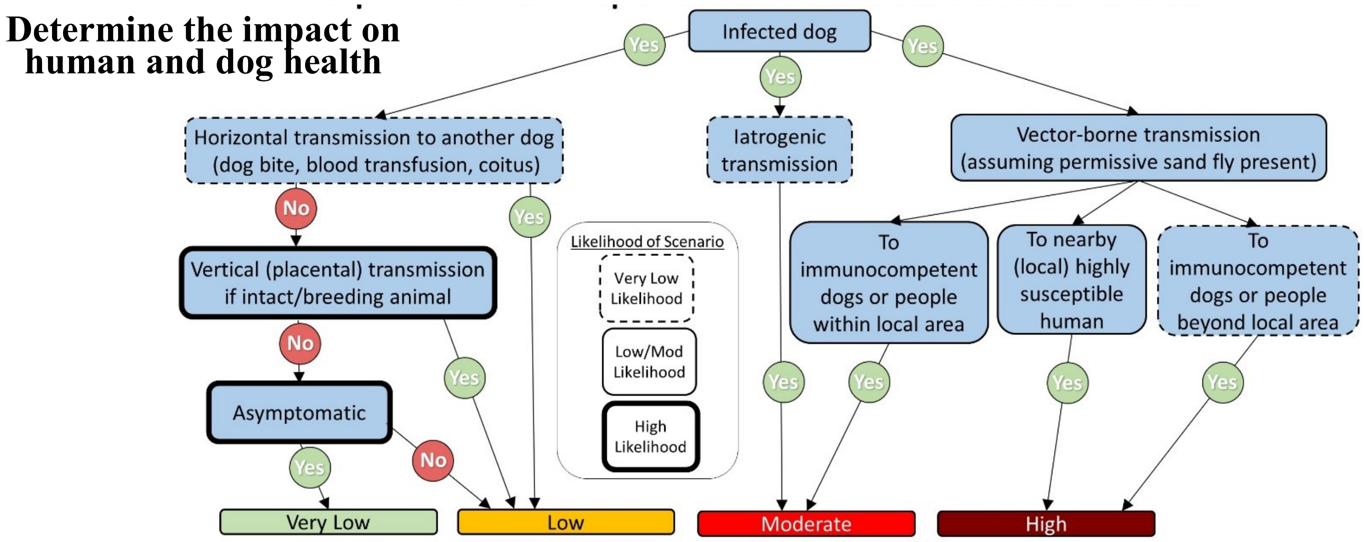

Step 5. Estimate final risk: use the final risk estimation matrix below to determine the *L. infantum* importation risk in dogs from endemic countries based on likelihood of importation and vector borne transmission and it's consequences on human and dog health.

**Combined importation & transmission probability**	**Impact of *L. infantum* transmission on dog and human health**			
	Very Low	Low	Moderate	High
Very Low	**Negligible Risk**	**Negligible Risk**	**Very Low Risk**	**Low Risk**
Low	**Negligible Risk**	**Very Low Risk**	**Low Risk**	**Moderate Risk**
Moderate	**Very Low Risk**	**Low Risk**	**Moderate Risk**	**High Risk**
High	**Very Low Risk**	**Low Risk**	**Moderate Risk**	**High Risk**
